# Inhibitory effects of superoxide dismutase 3 on *Propionibacterium acnes*-induced skin inflammation

**DOI:** 10.1038/s41598-018-22132-z

**Published:** 2018-03-05

**Authors:** Cuong Thach Nguyen, Shyam Kishor Sah, Christos C. Zouboulis, Tae-Yoon Kim

**Affiliations:** 1 Department of Dermatology, College of Medicine, The Catholic University of Korea, Seoul, 137-040 South Korea; 2 Departments of Dermatology, Venereology, Allergology and Immunology, Dessau Medical Center, Brandenburg Medical School Theodor Fontane, Dessau, Germany

## Abstract

*Propionibacterium acnes* is a well-known commensal bacterium that plays an important role in the pathogenesis of acne and chronic inflammatory skin disease. In this study, we investigated the effect of superoxide dismutase 3 (SOD3) on *P*. *acnes*- or peptidoglycan (PGN)-induced inflammation *in vitro* and *in vivo*. Our data demonstrated that SOD3 suppressed toll-like receptor-2 (TLR-2) expression in *P*. *acnes-* or PGN-treated keratinocytes and sebocytes. Moreover, we found that SOD3 suppressed the expressions of phosphorylated nuclear factor-κB (NF-κB) and p38 in *P*. *acnes-* or PGN-treated cells. SOD3 also exhibited an anti-inflammatory role by reducing the expression of inflammasome-related proteins (NLRP3, ASC, caspase-1) and inhibiting the expression of pro-inflammatory cytokines, including tumor necrosis factor-α, interleukin-1β, interleukin-6, and interleukin-8. In addition, SOD3 reduced lipid accumulation and expression of lipogenic regulators in *P*. *acnes*-treated sebocytes. Recombinant SOD3-treated wild-type mice and SOD3 transgenic mice, which were subcutaneously infected with *P*. *acnes*, showed tolerance to inflammation through reducing inflammatory cell infiltration in skin, ear thickness, and expression of inflammatory mediators. Our result showed that SOD3 could suppress the inflammation through inhibition of TLR2/p38/NF-κB axis and NLRP3 inflammasome activation. Therefore, SOD3 could be a promising candidate for treatment of *P*. *acnes*-mediated skin inflammation.

## Introduction

*Propionibacterium acnes* (*P*. *acnes*) is an anaerobic, gram-positive bacterium that is frequently found on the human skin^1^. Growing evidence shows that this bacterium is an opportunistic pathogen, causing implant-associated infections including breast implants, neurosurgical shunts, cardiovascular devices, ocular implants, and prosthetic joints^2,3^. *P*. *acnes* is responsible for the development of acne vulgaris, a common inflammatory skin diseases^4^. In keratinocytes, *P*. *acnes* produces reactive oxygen species (ROS) and triggers an inflammatory response^5^. Previous studies report that *P*. *acnes* activates TLR-2 to induce the production of pro-inflammatory cytokines, such as interleukin (IL)-1β and tumor necrosis factor-α (TNF-α) in keratinocytes, sebocytes, and monocytes^6–8^. Moreover, nuclear factor (NF)-κB, activator protein (AP)-1, pro-inflammatory cytokines (TNF-α, IL-1β, IL-6, and IL-8), and metalloproteinases (MMP) are activated via upregulation of TLR-2 in facial acne lesions of patients^9^. In macrophages, *P*. *acnes-*stimulated ROS triggers extracellular signal-related kinase (ERK), Janus kinase (JNK), NF-κB, and AP-1 activation, which induces expression of inducible NO synthase (iNOS)/NO and cyclooxygenase-2/prostaglandin E2 (COX-2/PGE2) during the infection^10^. In human sebocytes, *P*. *acnes* stimulates NLRP3 inflammasome, which regulates maturation and secretion of IL-1β and IL-18 via caspase-1 activity^11^. As a result, NLRP3-deficient mice show less inflammatory cytokine production, compared with wild-type mice^11^.

Superoxide dismutases (SODs) are antioxidant enzymes that function to dismutase two superoxide radicals to hydrogen peroxide and oxygen^12^. There are three isoforms of SOD in mammalian cells: cytoplasmic Cu/ZnSOD (SOD1), mitochondrial MnSOD (SOD2), and extracellular Cu/ZnSOD (SOD3)^12^. Mice lacking SOD3 function exhibit the development of inflammatory and fibrotic lung diseases^13^. SOD3 inhibits ovalbumin-induced allergic airway inflammation^14^, pathogenesis of chronic obstructive pulmonary disease (COPD)^13^, and ischemic injuries^15^. In addition, SOD3 suppresses pulmonary emphysema by inhibition of ECM fragmentation-induced oxidative stress^13^. Subcutaneous injection of allogeneic SOD3-transduced mesenchymal stem cells (MSCs) significantly suppresses imiquimod-induced psoriasis-like skin inflammation through inhibition of signaling pathways such as TLR-7, p-NF-κB, MAPKs, and adenosine receptor activation^16^. Furthermore, SOD3 reduces IL-23-mediated skin inflammation by inhibiting activation of immune responses and by suppressing infiltration of immune cells in mice^17^. SOD3 is shown to suppress hyaluronic acid fragments-mediated skin inflammation by inhibiting TLR-4 signaling pathway and blocking recruitment of NF-κB to the promoters of inflammatory genes^18^. Furthermore, SOD3 suppresses hypoxia-induced expression of angiogenic factors and pro-inflammatory cytokines through down-regulation of HIF-1α, PKC, and NF-κB signaling *in vitro* and *in vivo*^19^. These results showed that SOD3 has an important role in inflammatory diseases and this led us to investigate the role of SOD3 in *P*. *acnes*-induced skin inflammation.

In this study, we showed that *P*. *acnes* induced TLR-2 expression in a time- and dose-dependent manner at the mRNA levels. Moreover, SOD3 inhibited TLR-2 expression in keratinocytes and sebocytes treated with *P*. *acnes* or PGN. Further, SOD3 suppressed the expressions of p-NF-κB, p-p38, inflammasome proteins (NLRP3, ASC, and caspase-1), and inflammatory cytokines such as TNF-α, IL-1β, IL-6, IL-8 in *P*. *acnes-* or PGN-treated keratinocytes and sebocytes. Interestingly, SOD3 reduced lipid content and expression of lipogenic regulators in *P*. *acnes*-treated sebocytes. In a mouse model, treatment with SOD3 significantly inhibited *P*. *acnes*-induced skin inflammation including ear thickness, erythema, and infiltration of inflammatory cells, suggesting that SOD3 has anti-inflammatory effects in *P*. *acnes*-induced acne vulgaris.

## Results

### SOD3 inhibited TLR-2 and its downstream regulators in *P*. *acnes-* or PGN–treated HaCaT and SZ95 cells

**Figure 1:**
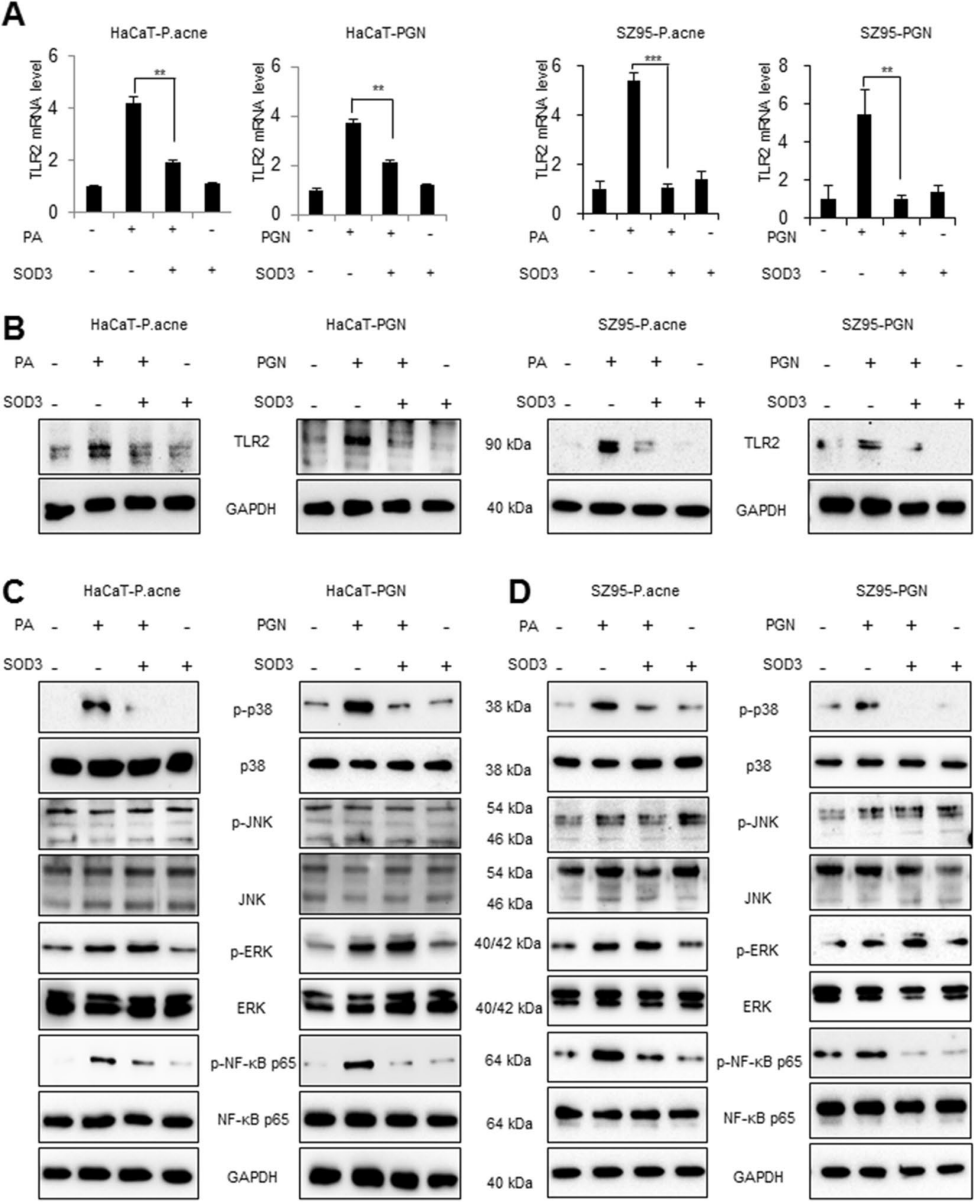
SOD3 inhibited expression of TLR2 and its downstream regulators in HaCaT and SZ95 cells. (**A**,**B**) HaCaT and SZ95 cells were pretreated with SOD3 (200 U/mL) and incubated with heat-killed *P*. *acnes* (100 MOI) or peptidoglycan (10 μg/mL) for 24 h. Expression of TLR-2 was analyzed by qRT-PCR (**A**) and Western blot (**B**). (**C**) HaCaT and SZ95 (**D**) cells were pretreated with SOD3 (200 U/mL) and incubated with heat-killed *P*. *acnes* (100 MOI) or peptidoglycan (10 μg/mL) for 24 h. Expression of p-NF-κB and MAPK proteins were analyzed by Western blot. Band density of Western blot data is shown in Supplemental Fig. S5A. Full-length blots are presented in Supplementary Fig. S6. All data represent the mean ± S.D. of three independent experiments *p < 0.05, **p < 0.01, ***p < 0.001.

TLR-2 expression is found to be up-regulated in clinical acne lesions^20^. Thus, we examined whether *P*. *acnes* or peptidoglycan (PGN control) activated TLR-2 and its downstream NF-κB signaling in HaCaT and SZ95 cells. As shown in Fig. S1A,B, *P*. *acnes*/PGN induced the expression of TLR-2 in a multiplicity of infection (MOI)-/dose- and time-dependent manner in HaCaT and SZ95 cells at the mRNA levels, compared with control (Fig. S1A,B). Based on this results and previous studies^21^, 100 MOI of heat-killed *P*. *acnes* or 10 μg/mL of PGN were used in our experimental settings. To further investigate the role of SOD3 on TLR-2 expression *in vitro*, we pre-treated the cells with SOD3 (200 U/mL) for an hour and then treated with *P*. *acnes* or PGN and examined TLR-2 expression. We found that SOD3 significantly decreased the expression of TLR-2 at the mRNA (Fig. 1A) and protein levels (Figs 1B and S1C,S5A) in both cells.

NF-κB and mitogen-activated protein kinases (MAPKs) are downstream regulators of the TLR-2 pathway. Thus, we aimed to evaluate whether SOD3 can also inhibit the activation of these regulators in *P*. *acnes-* or PGN-treated HaCaT and SZ95 cells. Our results showed that *P*. *acnes*/PGN induced significant phosphorylation of NF-κB in keratinocytes and sebocytes. Interestingly, SOD3 treatment suppressed the phosphorylation of NF-κB induced by *P*. *acnes* or PGN in both cells. In this study, the phosphorylation of p38 induced by *P*. *acnes* or PGN was inhibited in the SOD3-treated cells. Moreover, *P*. *acnes* or PGN increased phosphorylated ERK in the cells. However, effect of SOD3 on phosphorylation of ERK was not significant (Figs 1C,D and S5A). Treatment of *P*. *acnes* and SOD3 did not have any effects on phosphorylation of JNK.

In order to confirm the effects of recombinant SOD3 on expression of TLR2 and phosphorylation p38 and NF-κB, we knock-down expression of SOD3 and checked the expression of TLR2, p-38 and, p-NF-κB. Our results showed that knockdown of SOD3 slightly increased expression of TLR2 and phosphorylation of p38 and p-NF-κB in sebocytes but not in keratinocytes (Fig. S2).

### SOD3 decreased the expression of NLRP3 inflammasome in *P*. *acnes-* or PGN–treated HaCaT and SZ95 cells

**Figure 2:**
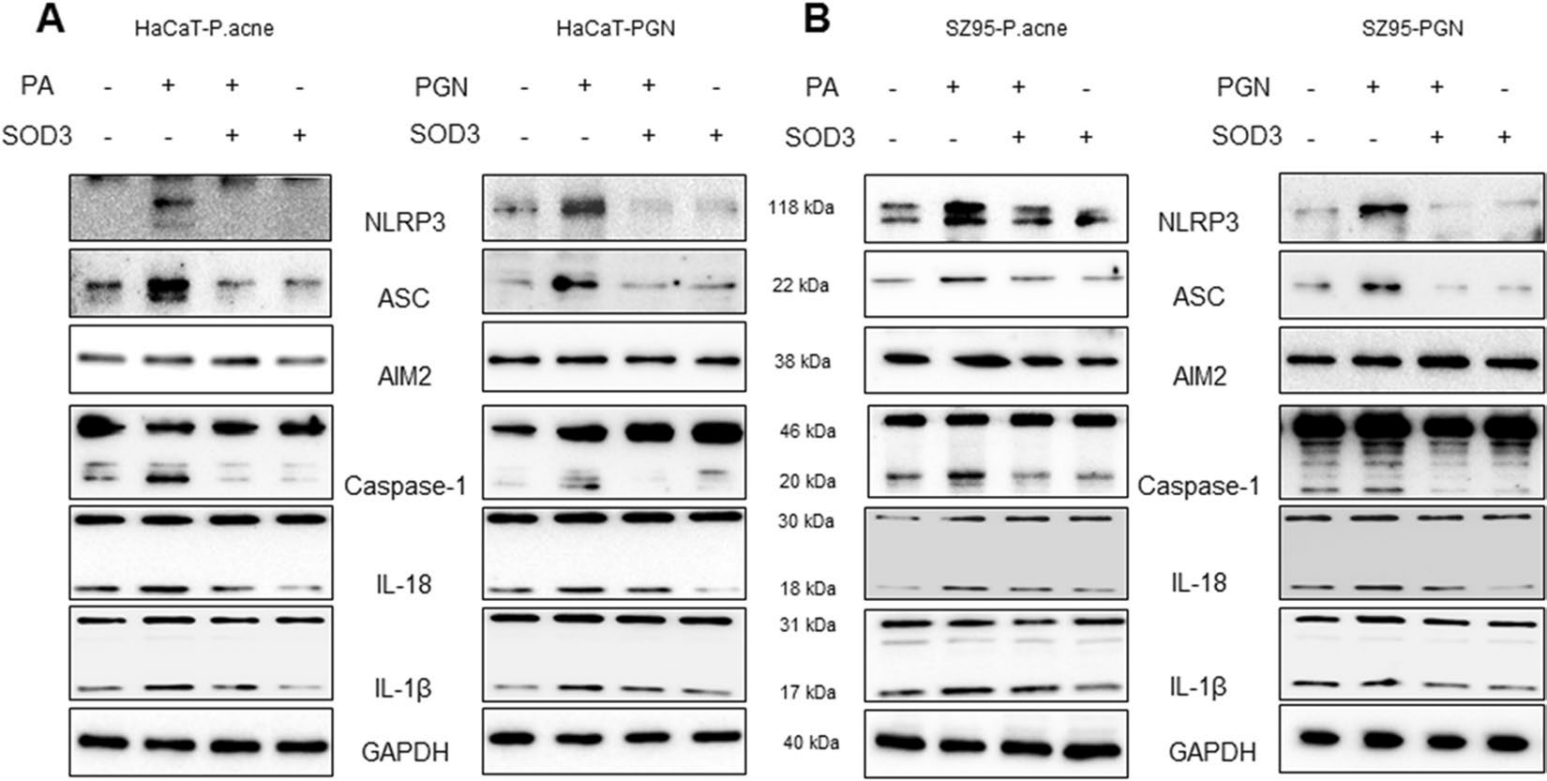
SOD3 inhibited *P*. *acnes*- or PGN-induced inflammasome in HaCaT and SZ95 cells. (**A**) HaCaT and SZ95 (**B**) cells were pretreated with SOD3 (200 U/mL) and incubated with heat-killed *P*. *acnes* (100 MOI) or peptidoglycan (10 μg/mL) for 24 h. Expression of inflammasome-related proteins was analyzed by Western blot. Full-length blots are presented in Supplementary Fig. S6. All data represent three independent experiments. Band density of Western blot data is shown in Supplemental Fig. S5B.

A previous study showed that *P*. *acnes* triggers expression of inflammasome and modulates inflammatory cytokine production^21^. Thus, we investigated the effects of SOD3 on activation of NLRP3 and expression of inflammasome**-**related proteins in *P*. *acnes*- or PGN-treated HaCaT and SZ95 cells. As shown in Fig. 2, *P*. *acnes* or PGN induced expression of NLRP3 in both cells, whereas SOD3 effectively inhibited the *P*. *acnes*- or PGN-induced expression of NLRP3 (Figs 2A,B and S5B). Moreover, SOD3 also reduced the *P*. *acnes*- or PGN-induced expression of ASC protein but did not have an effect on AIM2 expression. Consistently, maturation of caspase-1 was significantly induced by *P*. *acnes* or PGN, whereas treatment with SOD3 abolished the caspase-1 maturation. Activation of caspase-1 is important for proteolytic cleavage of mature IL-1β and IL-18. Thus, we examined whether SOD3 treatment could also regulate maturation of IL-1β and IL-18. The data showed that *P*. *acnes/*PGN significantly activated the expression of matured IL-1β and IL-18 at the protein level, whereas SOD3 treatment inhibited their maturation (Figs 2A,B and S5B).

In order to confirm the effects of recombinant SOD3 on expression of NLRP3 and ASC, we knockdown SOD3 expression and checked the levels of NLRP3 and ASC. Figure S2 showed that knockdown of SOD3 slightly enhanced expression of NLRP3 and ASC in sebocytes and keratinocytes.

### SOD3 effectively inhibited *P*. *acnes*- or PGN-induced pro-inflammatory cytokines *in vitro*

**Figure 3:**
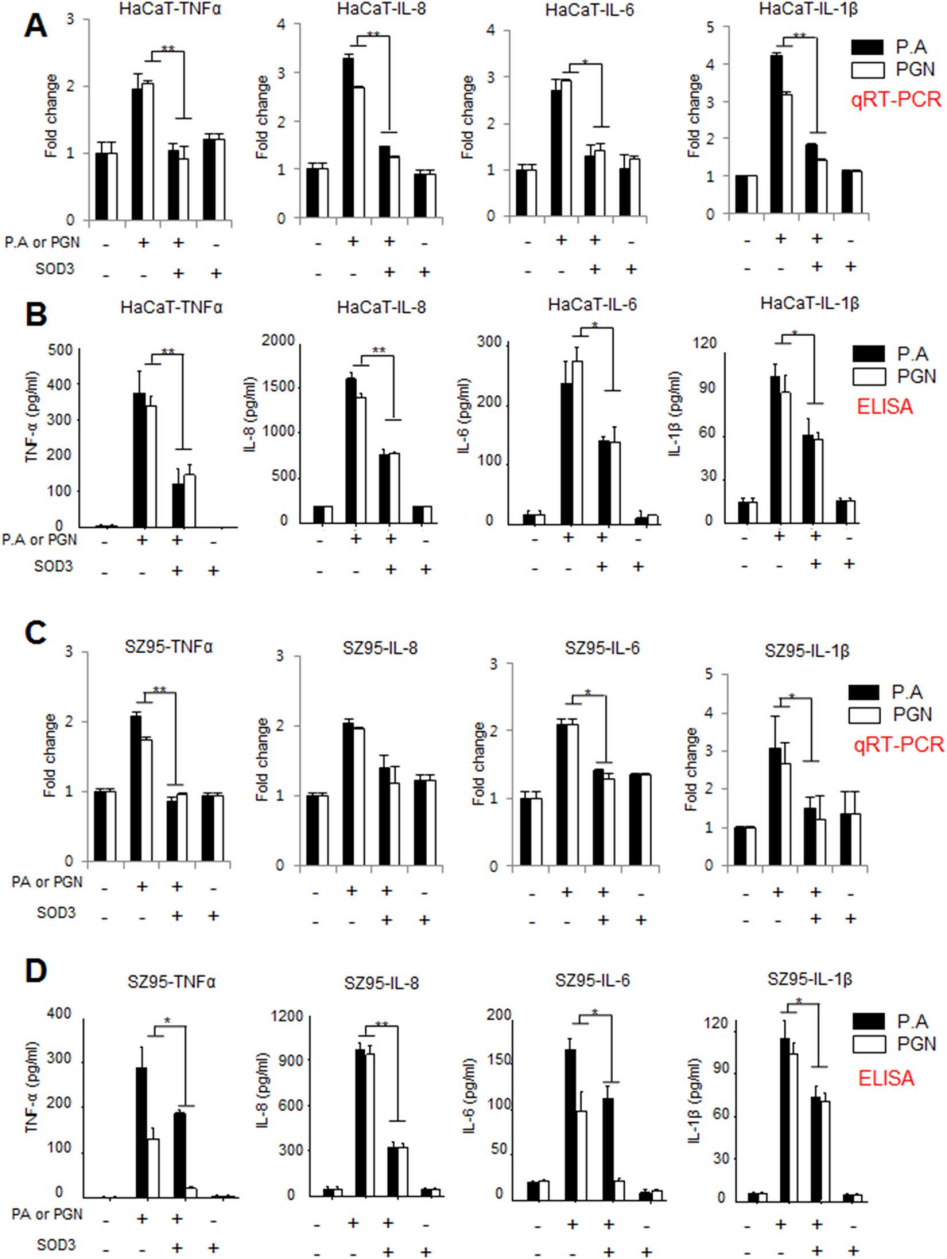
SOD3 reduced the levels of pro-inflammatory cytokines in *P*. *acnes*- or PGN-treated HaCaT and SZ95 cells. HaCaT (**A**,**B**) and SZ95 (**C**,**D**) cells were pretreated with SOD3 (200 U/mL) and incubated with heat-killed *P*. *acnes* (100 MOI, black bars) or peptidoglycan (10 μg/mL, white bars) for 24 h. Expression of inflammatory cytokines was analyzed by qRT-PCR (**A**,**C**) and ELISA (**B**,**D**). All data represent the mean ± S.D. of three independent experiments *p < 0.05, **p < 0.01, ***p < 0.001.

We have shown that SOD3 suppressed TLR-2 and inflammasome expression, which modulates the expression of inflammatory cytokines in *P*. *acnes*- or PGN-treated cells. Pro-inflammatory cytokines such as TNF-α, IL-1β, IL-6, and IL-8 are responsible for the follicular hyper-keratinization and inflammatory characteristic of acne^22^. Thus, we studied the effect of SOD3 treatment on the expression of these pro-inflammatory mediators *in vitro*. Our data showed that *P*. *acnes* or PGN induced the expression of pro-inflammatory mediators at the mRNA and protein level in HaCaT and SZ95 cells (Fig. 3A–D). Interestingly, qRT-PCR and ELISA results showed that SOD3 suppressed significantly the expression of these pro-inflammatory cytokines in both cells. These data suggested that SOD3 reduced *P*. *acnes*/PGN-induced inflammation *in vitro*.

### SOD3 impaired lipid accumulation in *P*. *acnes*- or PGN-treated SZ95 cells

**Figure 4:**
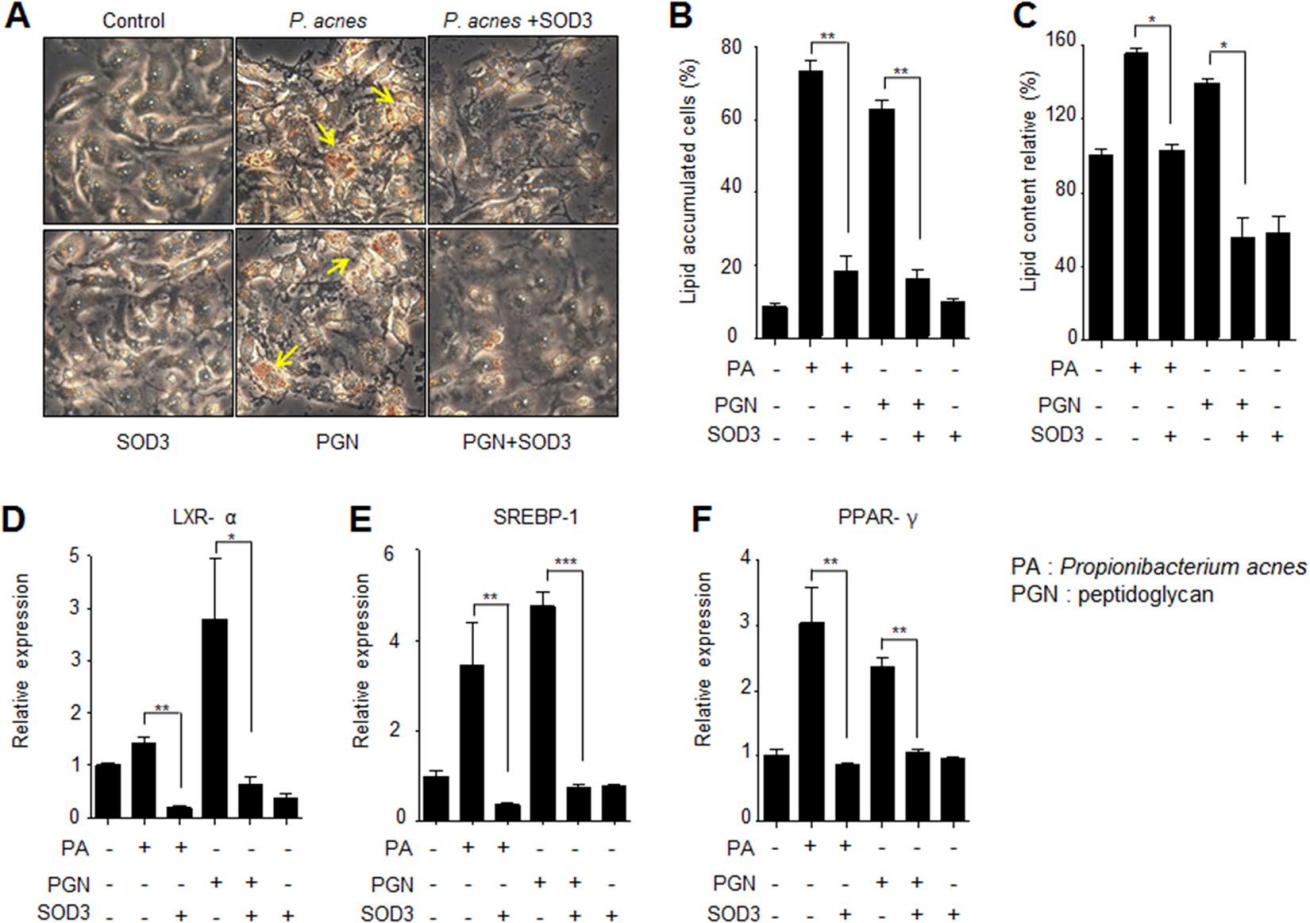
Effect of SOD3 on lipid accumulation in *P*. *acnes*-infected sebocytes SZ95. (**A**,**B**) SZ95 cells were pretreated with SOD3 (200 U/mL) and incubated with heat-killed *P*. *acnes* (100 MOI) or peptidoglycan (10 μg/mL) for 24 h. Lipid accumulation was determined by Oil Red O staining. (**C**) Quantification of lipid accumulation positive sebocytes. (**D**–**F**) Expression of LXR-α, PPAR-γ, and SREBF-1 was examined by qRT-PCR. All data represent the mean ± S.D. of three independent experiments *p < 0.05, **p < 0.01, ***p < 0.001.

*P*. *acnes*-induced lipogenesis is one of the important steps of acne vulgaris development^23^. To investigate the effects of SOD3 in lipid synthesis in *P*. *acne*- or PGN-treated sebocytes, we examined intracellular lipid accumulation in *P*. *acnes*- or PGN-treated sebocytes. Our results showed that *P*. *acnes*/PGN induced lipid accumulation in sebocytes whereas SOD3 treatment markedly reduced the lipid accumulation, compare with control (Fig. 4A–C).

Liver X receptor (LXR)-α, peroxisome proliferator-activated receptor (PPAR)-γ, and sterol regulatory element-binding transcription factor (SREBF)-1 are crucial regulators of lipogenesis^24^. To determine whether SOD3 inhibited the expression of these lipogenic regulators, we performed qRT-PCR to examine the expression levels of these genes in *P*. *acnes*- or PGN-treated sebocytes. Our data showed that *P*. *acnes*/PGN increased expression of LXR-α, PPAR-γ, and SREBF-1 in sebocytes compared to the control, whereas SOD3 treatment reduced expression of these genes (Fig. 4D–F), indicating that SOD3 inhibited expression of lipogenic regulators and lipid accumulation in sebocytes.

### SOD3 suppressed *P*. *acnes*-mediated skin inflammation in mice

**Figure 5:**
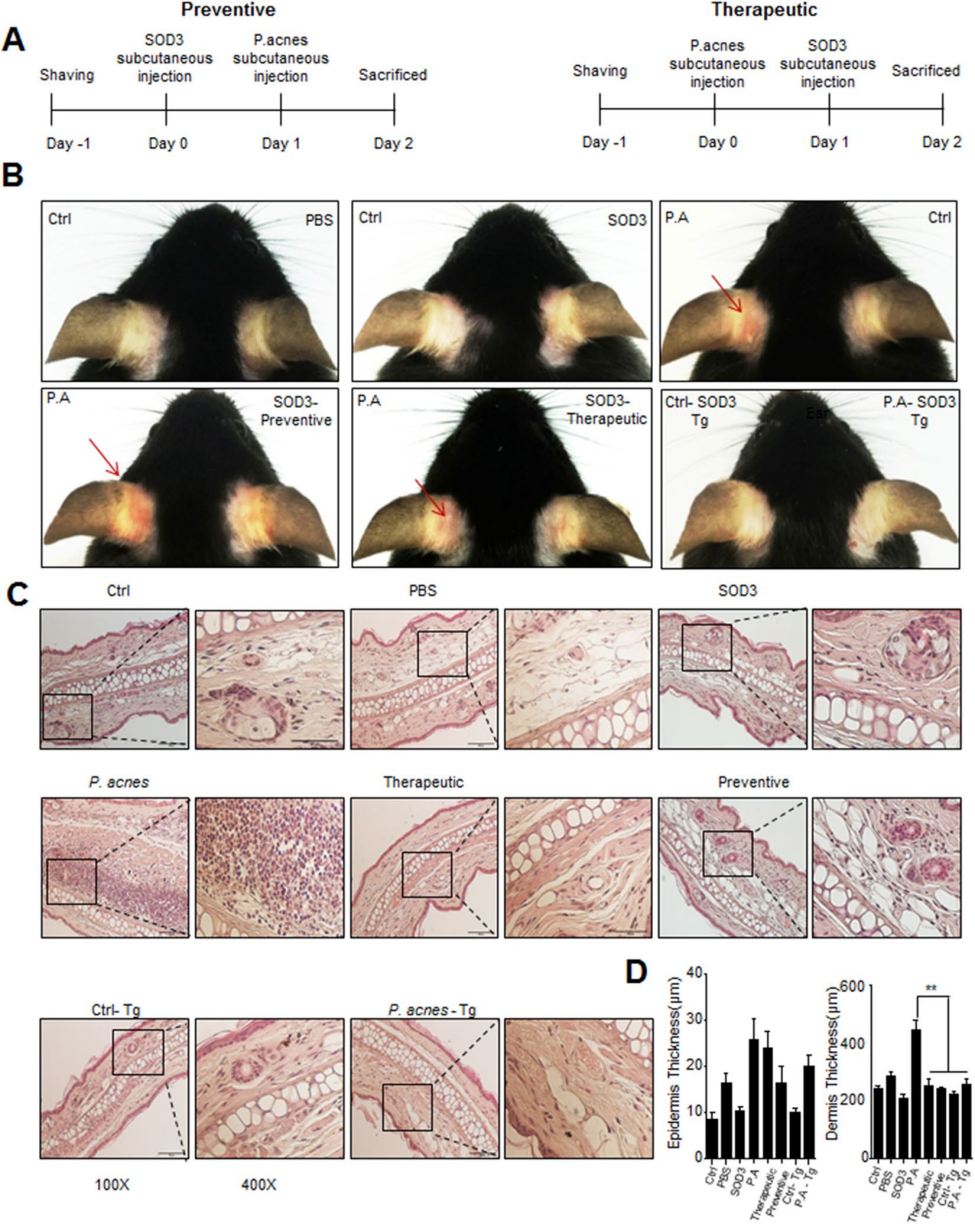
SOD3 reduced inflammation and ear thickness in *P*. *acnes*-injected mice. (**A**) Experimental scheme for *in vivo* studies. (**B**) Ear inflammation phenotype. (**C**) Representative HE staining images of each group (100×, 400×). (**D**) Ear, epidermis and dermis thickness. All data represent the mean ± S.D. of three independent experiments *p < 0.05, **p < 0.01, ***p < 0.001.

To investigate the role of SOD3 in *P*. *acnes*-induced skin inflammation *in vivo*, mice were subcutaneously injected with *P*. *acnes* and human recombinant SOD3 on day 0 or 1 and were sacrificed on day 2 (Fig. 5A). Phenotypically, at 12 h post-injection, *P*. *acnes*-injected mice started to display a sign of inflammation, such as erythema, which continuously increased in severity up to the end of the experiment, but neither symptom was observed in PBS-injected mice (Fig. 5B). Interestingly, pre- and post-treatment of SOD3 showed significantly less erythema compared with the *P*. *acnes*-treated group (Fig. 5B). H&E staining data showed increased dermal thickness and infiltration of inflammatory cells into the dermis with the *P*. *acnes*-treated group. In contrast, SOD3-treated and SOD3 Tg mice showed a significant decrease in ear thickness and reduced infiltration of inflammatory cells in comparison with *P*. *acnes*-treated mice (Fig. 5C,D).

### SOD3 inhibited expression of TLR-2/p38/NF-κB axis and inflammatory mediators in mice

**Figure 6:**
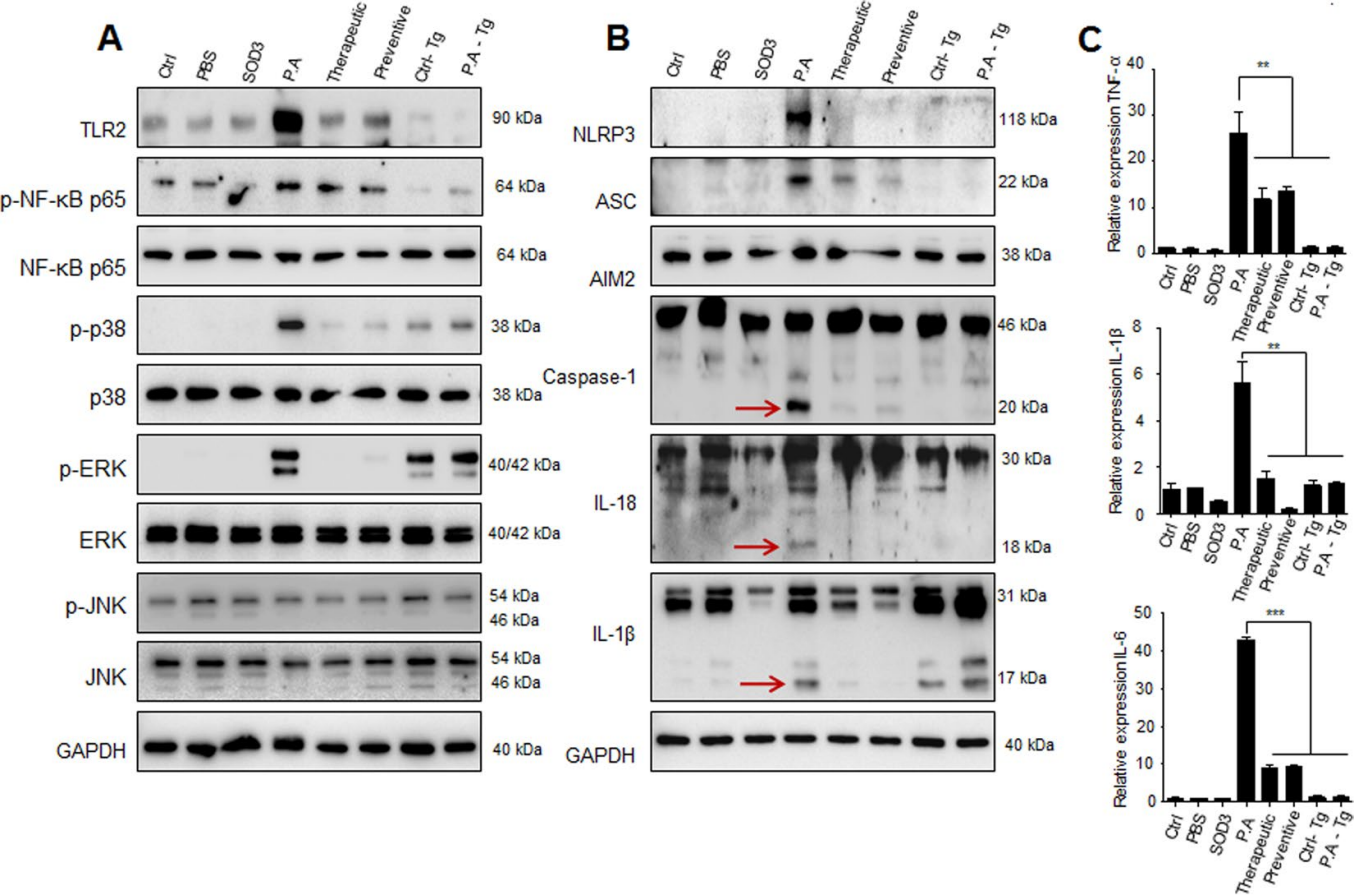
SOD3 inhibited inflammatory mediators *in vivo*. (**A**,**B**) Proteins were isolated from ears and the protein expressions were determined by Western blot with the indicated antibodies. Arrows indicate the mature form of caspase-1, IL-18, and IL-1β. Band density of Western blot data is shown in Supplemental Figure S5C. Full-length blots are presented in Supplementary Figure S6. (**C**) Expression of the cytokines was examined by qRT-PCR. All data represent the mean ± S.D. of three independent experiments *p < 0.05, **p < 0.01, ***p < 0.001.

We further investigated the effects of SOD3 on the expression of TLR-2, p-NF-κB, and p-p38 in mice. As shown in Fig. 6A, mice injected with *P*. *acnes* showed increased expression of TLR-2, p-NF-κB, and p-p38 at the protein level, whereas SOD3-treated and SOD3 Tg mice showed lower levels of TLR-2, p-NF-κB, and p-p38 expression (Figs 6A and S5C). Moreover, expression of TLR-2 and p-NF-κB was lower in SOD3 Tg mice, compared with SOD3-treated mice. In addition, *P*. *acnes*–induced phosphorylated ERK was abolished in recombinant SOD3-treated mice whereas the phosphorylated ERK was not difference between control Tg mice and *P*. *acnes*-treated Tg mice. Furthermore, *P*. *acnes*-injected mice showed high-level expression of inflammasome related proteins (NLRP3, ASC, caspase-1) and maturation of IL-1β and IL-18, whereas SOD3-treated and SOD3 Tg mice exhibited lower protein levels of these inflammasome markers (Figs 6B and S5C). These data suggest that SOD3 suppresses the *P*. *acnes*-induced TLR-2, p-NF-κB, p-p38, and inflammasome-related proteins expression *in vivo*.

Next, we studied the effect of SOD3 treatment on the expression of proinflammatory cytokines in the skin of *P*. *acnes*-injected mice. Consistent with the histological changes, qRT-PCR analysis also showed that the mRNA levels of inflammatory cytokines (TNF-α, IL-1β, and IL-6) were increased in the *P*. *acnes*-treated skin, whereas SOD3-treated and SOD3 Tg mice have lower levels of inflammatory cytokines, compared with control (Fig. 6C), suggesting that SOD3 inhibited the *P*. *acnes*-induced skin inflammation in mice.

## Discussion

Oxidative stress is considered one of important factors in inflammatory skin diseases such as acne vulgaris, atopic dermatitis, and psoriasis. A recent study showed that RoxP, a secreted antioxidant enzyme produced by *P*. *acnes*, might be beneficial for the bacterium and the host^25^. RoxP binds to heme and reduces the level of free radicals, thus protecting biomolecules from oxidation. Although *P*. *acnes* is a commensal bacterium of normal skin flora, several *P*. *acnes* strains associated with acne patients have been identified and considered to play an important role in acne development^26^. The genomic analysis showed that virulent *P*. *acnes* strains produce some virulence factors such as sialidases, neuraminidases, endoglycoceramidases, lipases, and hemolysins, which destroy the host tissue and play major impact on the acne pathogenesis^27,28^. In addition, *P*. *acnes* strain (KCTC 3314) used in this study has been reported to have a role in skin inflammation and acne pathogenesis^11^. Upon *P*. *acnes* infection, ROS, and especially superoxide anions (O2•−), were rapidly produced by skin cells to response the infection^5^. The ROS initiates to trigger the inflammation in skin. Clinically, major pathophysiological features of acne include hyper-keratinization, androgen-stimulated sebaceous secretion, obstruction of sebaceous follicles, proliferation of *P*. *acnes*, and inflammation^29^. In clinical acne samples, *P*. *acnes* triggers activation of TLR-2 which modulates production of inflammatory cytokines, including TNF-α, IL-1β, IL-6, and IL-8^7,30^. Moreover, inhibition of TLR-2 activation reduces the production of inflammatory cyokines induced by *P*. *acnes* infection *in vitro*^7,8,30^. Sebocytes and keratinocytes are major components of the pilosebaceous unit and act as immune cells activated by *P*. *acnes* via TLRs^31^. In this study, we showed that SOD3 treatment suppressed the expression of TLR-2 and cytokines TNF-α, IL-1β, IL-6 and IL-8, which were induced by *P*. *acnes* or PGN in keratinocytes and sebocytes. Moreover, it should be noted that we used PGN from *S*. *aureus* instead of from *P*. *acnes* to induce inflammatory response in this study because PGN from *P*. *acnes* is not commercially available. *S*. *aureus* was considered to be associated with skin inflammatory diseases including acne^32,33^. Thus, our results suggested that SOD3 may inhibit skin inflammation-induced by either *P*. *acnes* or possibly by *S*. *aureus*.

*P*. *acnes*-activated TLR-2 triggers the activation of NF-κB and MAPKs signaling pathways, which are responsible for inflammatory cytokine production and the innate immune response^6^. Activation of NF-κB and p38 have been found in clinical acne lesions^34,35^, indicating that NF-κB and MAPKs signaling are important for the pathogenesis of acne vulgaris. Therefore, suppression of p38 MAPK and NF-κB activation exhibits a protective effect on skin inflammation^34^. Our results showed that SOD3 reduced the phosphorylation of p38 and NF-κB in cells and in mice treated with *P*. *acnes*. In addition, treatment of recombinant SOD3 suppressed phosphorylation of ERK in *P*. *acnes*-treated mice but SOD3 Tg mice did not show any inhibitory effect on the ERK phosphorylation, compared with control Tg mice. This distinct effect could be explained based on the difference in genetic background of C57BL/6 mice and SOD3 Tg mice.

In human sebocytes infected with *P*. *acnes*, the NRLP3 inflammasome level is shown to be increased, leading to activation of capase-1 activity, which cleaves the precursors of IL-1β and IL-8^21^. Moreover, ASC is responsible for sensing the presence of *P*. *acnes* then inducing maturation of IL-1β. The cleavage of precursor IL-1β and IL-8 is one of the crucial steps in the secretion of IL-1β and IL-8^11^. *P*. *acnes* infection triggers strong inflammatory responses and NLRP3-inflammasome *in vivo*^11^. In addition, bone marrow–derived dendritic cells (BMDC) from caspase-1-deficient mice do not have the ability to produce IL-1β during *P*. *acnes* infection^21^. Our data showed that SOD3 reduced the expression of the inflammasome proteins (NLRP3, ASC, caspase-1), leading to the inhibition of IL-1β and IL-8 maturation in cells and mice upon *P*. *acnes* treatment.

We showed that recombinant SOD3 inhibited TLR-2/p-p38/p-NF-κB pathway and NLRP3 inflammasome. In order to confirm these observations, we examined effects of SOD3 knockdown on these signaling pathways in *P*. *acnes*-treated keratinocytes and sebocytes. Interestingly, knockdown of SOD3 slightly increased expression of TLR-2, p-p38 and p-NF-κB even without *P*. *acnes* treatment in both cells. In *P*. *acnes*-treated keratinocytes, expression of TLR-2, p-p38, and p-NF-κB was not much different between SOD3-knockdown keratinocytes and control cells (Fig. S2) whereas knockdown of SOD3 showed slightly increased expression level of NLRP3 and ASC, compared with control (Fig. S2). In addition, expression of TLR-2, p-p38, p-NF-κB, NLRP3, and ASC was increased in *P*. *acnes*-treated sebocytes, compared with control cells. These results suggested that SOD3 negatively regulated expression of TLR2/MAPKs/NF-κB axis and NLRP3 inflammasomes.

Frankel *et al*. discovered that the purified proteins could be taken up by cells and subsequently transactivate expression of cellular genes^36^. These proteins are considered as cell-penetrating peptides or protein transduction domains (PTDs) such as the HIV transactivator protein (TAT). Heparin binding domain (HBD) of SOD3 has been shown to have similar features as the other cell-penetrating peptides because of highly basic residues of the HBD domain. It has been reported that HBD of SOD3 acts as a signal domain for processes of secretion, re-uptake, and nuclear localization^37^. In previous study, our group demonstrated that heparin binding domain (HBD) is important for an uptake of recombinant SOD3 from media to cell in the presence of Zn^2+^, resulting in an accumulation of recombinant SOD3 in the nucleus^38^. This resulted in increased inhibitory effects of SOD3 on NF-kB and MAPKs signals. However, the intracellular uptake mechanism of SOD3 HBD peptide is not well understood and further studies need to elucidate the molecular mechanism(s).

*P*. *acnes* significantly induces cathelicidin (LL-37) in sebocytes and human β-defensin-2 (hBD2) in clinical samples^39^. Antimicrobial peptides, such as LL-37 and hBD2, were shown to be essential for skin protection against bacterial infection *in vivo*^40^. However, aberrant production of LL-37 and hBD2 causes inflammatory skin diseases, including atopic dermatitis, psoriasis, or rosacea. Thus, we examined whether SOD3 inhibited the levels of LL-37 and hBD2 expression during *P*. *acnes* infection. Our results indicated that *P*. *acnes* induced expression of LL-37 and hBD2 in both cell lines, while SOD3 treatment decreased the expression of LL-37 and hBD2 induced by *P*. *acnes* or PGN (Fig. S3), indicating that SOD3 also reduced LL-37 and hBD2 expression *in vitro*.

*P*. *acnes* induces SREBF-1 expression, which has an important role in lipogenesis in sebocytes^41^. Inflammatory processes play a major role in the development of acne vulgaris. Lipid accumulation could promote acne vulgaris because oxidation of lipids can stimulate production of inflammatory cytokines^42^. Our data indicated that SOD3 reduced expression of lipogenic regulators (LXR-α, PPAR-γ, and SREBF-1) in *P*. *acnes*- or PGN-treated sebocytes, thus diminishing lipid accumulation in *P*. *acnes*- or PGN-treated sebocytes.

Previously, our group observed that SOD3 inhibits dendritic cell (DC) maturation via the suppression of major histocompatibility complex (MHC) II, CD80, and CD86 expression; however, neither SOD1 nor SOD2 inhibits DC maturation^14^. Moreover, SOD3 decreases Th_2_ and Th_17_ differentiation, whereas both SOD1 and SOD2 do not show any effects on T cell differentiation^14^. Further, as shown in Figs S4 and S5D, SOD1 treatment did not inhibit the expression of p-NF-κB in HaCaT cells whereas SOD1 suppressed lightly phosphorylated p38 induced by *P*. *acnes-* or PGN. In addition, alone treatment of SOD1 induced expression of p-NF-κB in both cells, compared with control. The distinct effects between SOD3 and SOD1 could be explained based on their cellular localization and their half-lives of approximately 20 h for extracellular SOD3 and approximately 6 min for cytoplasmic SOD1^43^, indicating the specific role of SOD3 in modulation of the innate immune response to *P*. *acnes* infection. Role of SOD2 was not compared in this study because SOD1 and SOD3 use Zn/Cu as a catalytic metal cofactor whereas Mn^2+^ ion is necessary for SOD2 activity.

**Figure 7:**
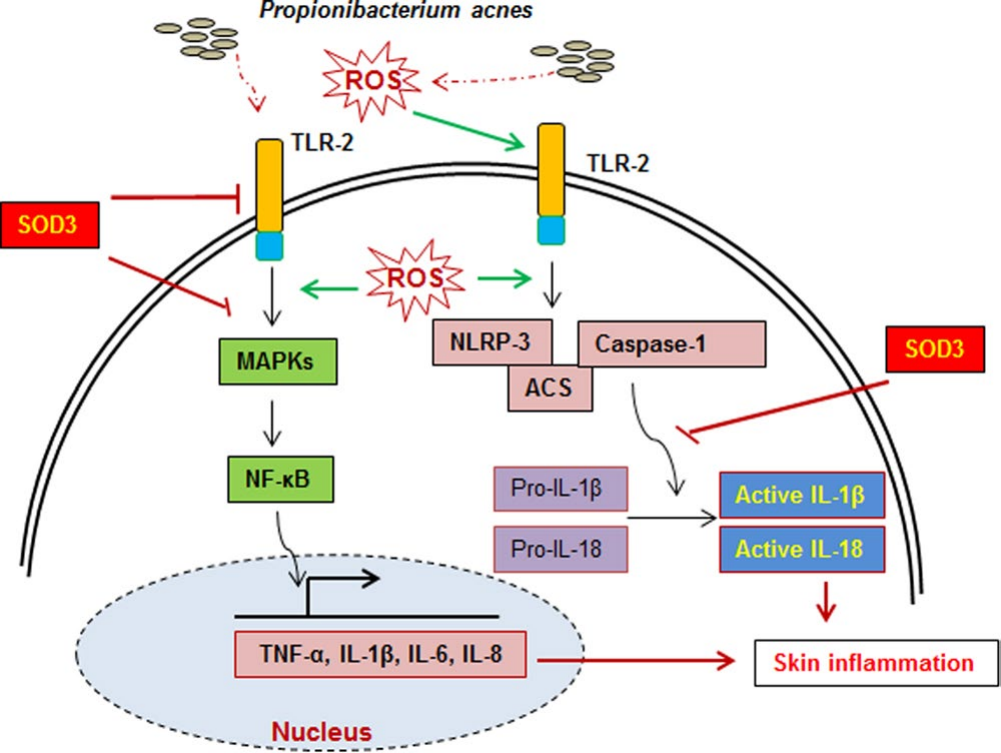
A proposed function of SOD3 on *P*. *acnes*-induced skin inflammation. During *P*. *acnes* infection, *P*. *acnes* triggered ROS production as previous reported, activation of TLR2/MAPKs/NF-κB axis, and expression of inflammasome components which modulated production of pro-inflammatory cytokines and skin inflammation. SOD3 inhibited skin inflammation through suppression of these signal pathways.

Based on our results and previous findings, we proposed a possible mechanism of SOD3 in *P*. *acnes*-mediated inflammation (Fig. 7). *P*. *acnes* triggers ROS production and activates TLR2^5^. *P*. *acnes* induced production of inflammatory cytokines (TNF-α, IL-1β, IL-6, and IL-8) through TLR-2/MAPKs and NLRP3 inflammasome. Our previous data showed that SOD3 inhibits the trafficking of TLR4 to lipid rafts, leading to inhibition of inflammation^18^. Thus, it is possible that SOD3 also inhibits *P*. *acnes*-induced TLR2 expression and trafficking of TLR2 to lipid rafts, leading to inhibit activation of NF-κB and MAPKs pathways. Moreover, *P*. *acnes*-induced ROS is necessary to trigger of NLRP3 inflammasome activation in sebocytes^11^. Here, SOD3 inhibits the expression of the NLRP3 inflammasome likely by reducing ROS level. As a result, SOD3 suppresses the inflammation via inhibition of TLR2/MAPKs/NF-κB and NLRP3 inflammasome.

Furthermore, several skin inflammatory diseases have been treated with steroids and/or antibiotics. However, using these drugs long-term has some unexpected side effects, such as hypertension, immuno-suppression, and antibiotics resistance. In addition, light- and laser-based treatment, which has shown to kill *P*. *acnes*, being used to treat acne vulgaris^44^. However, this method was not sufficient to inhibit the *P*. *acnes*-induced inflammation because heat-killed *P*. *acnes* is capable to induce the inflammation^45^. Our result showed that SOD3 expression was decreased by heat-killed *P*. *acnes* infection leading to inflammation (Fig. S4) and treatment of SOD3 reduced heat-killed *P*. *acnes*-mediated inflammation. Our *in vivo* results also showed that live *P*. *acnes* induced skin inflammation, which is similar to clinical features of acne, and SOD3 suppressed the live *P*. *acnes*-induced inflammation, suggesting that SOD3 could inhibit the skin inflammation induced by heat-killed and live *P*. *acnes in vitro* and *in vivo*, respectively. Thus, SOD3 could be a promising candidate for the treatment of skin inflammatory diseases. These results suggested that treatment of SOD3 could be an additional treatment for management of inflammatory skin diseases induced by *P*. *acnes*.

## Materials and Methods

### Mice and *P*. *acnes* injection

C57BL/6 mice and SOD3 Tg mice were used, as previously described^18^, and kept under specific pathogen-free conditions. Experimental procedures were approved and monitored by the Catholic Ethics Committee of the Catholic University of Korea, which conform to the National Institutes of Health guidelines.

Eight-week-old C57BL/6 mice (24 mice) were randomly divided into eight groups (three mice/group), including WT mice without injection, PBS control group (subcutaneous injection into the ear with 20 μl PBS), SOD3 control group (subcutaneous injection into the ear with 2000 U of SOD3), *P*. *acnes* group (subcutaneous injection with 1 × 10^7^ CFU of *P*. *acnes*), therapeutic group (subcutaneous injection with 1 × 10^7^ CFU of *P*. *acnes* then injection with 2000 U of SOD3), preventive group (subcutaneous injection with 2000 U of SOD3 then injection with 1 × 10^7^ CFU of *P*. *acnes*), SOD3 Tg mice without injection, SOD3 Tg mice injected with 1 × 10^7^ CFU of *P*. *acnes*. At the end of the experiment, mice were sacrificed and ears were sampled for H&E staining, qRT-PCR, and Western blot. Three independent experiments were performed.

### Reagents and antibodies

Peptidoglycan from *Staphylococcus aureus* cell wall component (PGN, 77140) was purchased from Sigma-Aldrich (MO, USA). TLR-2 (ab16894), IL-18 (ab71495) antibodies were purchased from Abcam (Cambridge, UK). p-NF-κB p65 (sc-101752), NF-κB p65 (sc-109), ASC (sc-22514), AIM2 (sc-137967, sc-515514), caspase-1 (sc-1597,), IL-18 (sc-7954), GADPH (sc-32233) antibodies were purchased from Santa Cruz Biotechnology (CA, USA). p-p38 (9211 S), p38 (9212 S), p-JNK (9251 S), JNK (5252 S) p-ERK (9101 S), ERK (9101), IL-1β (12242 S), NLRP3 (13158 S), caspase-1 (4199 S) antibodies were purchased from Cell Signaling Technology (MA, USA). Goat anti-Rabbit and anti-mouse IgG (H + L) Cross-Adsorbed Secondary Antibodies, HRP conjugate were purchased from Thermo Scientific (CA, USA). Lysis buffer (150 mM NaCl, 1.0% IGEPAL® CA-630, 0.5% sodium deoxycholate, 0.1% sodium dodecyl sulfate, 50 mM Tris, pH 8.0) was purchased from HNS Bio Co., Ltd (Seoul, Korea).

### Cell culture

The human HaCaT keratinocytes were purchased from Thermo Scientific (MA, USA) and SZ95 sebocytes were characterized as previously described^46^. HaCaT cells and SZ95 sebocyteswere grown in Dulbecco’s Modified Eagle’s Medium (DMEM) or SebomedTM basal medium (Biochrom, Berlin, Germany), respectively, supplemented with 10% fetal bovine serum (Lonza, Basel, Switzerland), 100 U/mL penicillin, and 100 μl/mL streptomycin at 37 °C in a humidified incubator under 5% CO_2_.

### Preparation of bacteria

*P*. *acnes* (KCTC 3314) was obtained from the Korean Collection for Type Cultures (KCTC, Daejeon, Korea) as pervious described^11^. Brain-heart infusion (BHI) agar (Oxoid, Basingstoke, UK) was used to growth *P*. *acnes* for 48–72 hours at 37 °C under anaerobic conditions (5% H_2_, 5% CO_2_, and 90% N_2_). The bacteria were inoculated into BHI broth until the culture reached the log-phase. The pellet was collected and heat-killed at 80 °C for 30 min.

### Western blot analysis

HaCaT and SZ95 cells (5 × 10^5^ cells per well) were seeded on six-well plate for 24 h and pre-treated with SOD3 (200 U/ml) for 1 h as previous described^17^ and subsequently treated with heat-killed *P*. *acnes* (100 MOI, 5 × 10^7^ CFU per well) or peptidoglycan (PGN, 10 μg/mL) for 24 h additional incubation. We did not observe any toxicity of SOD3 on the cells at concentration 200 U/ml. Cells were lysed in RIPA lysis buffer containing protease and phosphatase inhibitor cocktails (Sigma-Aldrich, MO, USA). Protein concentration was determined by the Bradford assay, as previously described^47^. Protein samples (50 μg) were separated by sodium dodecyl sulfate-polyacrylamide gel electrophoresis (SDS-PAGE) and transferred to polyvinylidene difluoride (PVDF) membranes. The membranes were blocked with 5% skim milk in Tris-buffered saline (TBS, pH 7.4) with 0.1% Tween 20 (TBST) for 2 h at room temperature and subsequently incubated with primary antibodies at 4 °C overnight. Membranes were then shaken in secondary antibodies conjugated with horseradish peroxidase (Invitrogen, Grand Island, NY, USA). The signals were detected with an Image-Analyzer System (PXi 4 EZ/PXi 4 EZ Touch, Syngene, UK). The data represent three independent experiments.

### RNA isolation and qRT-PCR

Total RNA was isolated from HaCaT, SZ95 cells or mouse skin using TRIzol reagent (Invitrogen). cDNA was synthesized from 1000 ng of total RNA using a reverse transcription system (Qiagen, Hilden, Germany).

The cDNA was used as the template for qRT-PCR. qRT-PCR was carried out with the LightCycler-DNA Master SYBR Green I kit (Roche Molecular Biochemicals). The primer sets for TLR-2, TNF-α, IL-8, IL-6, IL-1β, and GAPDH were purchased as the QuantiTect primer assay (Qiagen). GAPDH was used as an endogenous control.

### Enzyme-linked immunosorbent assay (ELISA)

TNFα, IL-6, IL-8, and IL-1β enzyme-linked immunosorbent assay (ELISA) kits were purchased from Biolegend. ELISA was performed according to the manufacturer’s instructions.

### SOD3 purification and activity assay

SOD3 was purified as previous described^38^. Briefly, SOD3 expression plasmid was transfected into HEK-293T cells (ATCC® CRL-11268™) with Attractene (Qiagen) based on the manufacturer’s instructions. Five days after transfection, culture media containing SOD3 were collected, filtrated, and loaded onto HiTrap Chelating HP column (GE Healthcare). After loading, the column was washed with more than 50 column volumes of washing buffer, 50 mM NaPO_4_, 500 mM NaCl, and 30 mM imidazole. Then, SOD3 was eluted by the elution buffer containing 50 mM NaPO_4_, 500 mM NaCl, 500 mM imidazole, followed by dialysis in PBS containing 50 μM Cu^2+^/Zn^2+^ ions. The concentration of purified SOD3 was determined based on a BSA standard curve with a protein assay dye (Bio-Rad). The recombinant SOD3 was verified by western blot with SOD3 antibody as previous described^43^.

The SOD3 enzymatic activity was measured by the Superoxide Dismutase Assay Kit-WST (Dojindo Molecular Technologies, MD, USA) following the manufacturer’s instructions. Briefly, a 20 µl sample or PBS (bank control) was mixed with 200 µl of 200 µM WST working solution and 20 µl of enzyme working solution. The mixtures were incubated for 20 min for developing the signal, which was read at A_450_ using a micro-plate reader. The SOD activity was determined from the dilution factor exhibiting 50% inhibition (IC_50_).

### Determination of lipid content

SZ95 human sebocytes (5 × 10^4^ cells/well) were cultured in a 6-well plate then treated with heat-killed *P*. *acnes* or peptidoglycan for 24 h. Cells were washed three times with PBS and fixed with 10% formaldehyde for 20 min. Fixed cells were washed twice with 60% isopropanol and stained with saturated 0.6% (w/v) Oil Red O staining solution (isopropanol: distilled water in a v/v ratio of 3:2) for 90 min. After washing three times with distilled water, Oil Red O-stained cells were observed using an optical microscope (Nikon, Tokyo, Japan). Oil Red O was extracted from the cells with 100% isopropanol and optical density (OD) was then measured at a wavelength of 520 nm.

### Hematoxylin and eosin (HE) staining

The ears were excised and immediately fixed with 4% paraformaldehyde at 4 °C for 48 h. Paraffin-embedded ears were cut into 4 μm cross-sections. The cross-sections were stained with hematoxylin and eosin (HE) and then visualized by a microscope in order to evaluate inflammatory cell infiltration.

### Statistical analysis

Statistical differences were analyzed by one-way ANOVA. All results represent three independent experiments. Statistically significant differences were considered as p < 0.05 (*p < 0.05, **p < 0.01, ***p < 0.001).

## Electronic supplementary material

Supplementary Materials
