# Author Correction: Inhibitory effects of superoxide dismutase 3 on *Propionibacterium acnes*-induced skin inflammation

**DOI:** 10.1038/s41598-018-31453-y

**Published:** 2018-08-29

**Authors:** Cuong Thach Nguyen, Shyam Kishor Sah, Christos C. Zouboulis, Tae-Yoon Kim

**Affiliations:** 1 Department of Dermatology, College of Medicine, The Catholic University of Korea, Seoul, 137-040 South Korea; 2 Departments of Dermatology, Venereology, Allergology and Immunology, Dessau Medical Center, Brandenburg Medical School Theodor Fontane, Dessau, Germany

Correction to: *Scientific Reports* 10.1038/s41598-018-22132-z, published online 05 March 2018.

Christos C. Zouboulis was omitted from the author list in the original version of this Article.

The Author Contributions section now reads:

“C.T.N. and T.Y.K. conceived the study. C.T.N. performed experiments. C.T.N., S.K.S. and T.Y.K. analyzed data, edited the manuscript. C.T.N. wrote manuscript. C.C.Z. provided the human sebaceous gland cell line SZ95. All authors discussed the results and reviewed the manuscript.”

The Acknowledgements section now reads:

“This work was supported by the Bio and Medical Technology Development Program, the National Research Foundation (NRF) funded by Korean Government, The Republic of Korea, MSIP (Grant No. NRF-2016M3A9B6903020) and by the Ministry of Science, ICT & Future Planning (Grant No. NRF-2013M3A9A9050567), and the Industrial Technology Innovation program (10063322, Development of Animal Cell Culture based hEC-SOD for Management of Atopic Dermatitis) funded By the Ministry of Trade, industry & Energy.”

Additionally, the following reference was omitted:

47. Zouboulis, C. C., Seltmann, H., Orfanos, C. E. & Neitzel, H. Establishment and characterization of an immortalized human sebaceous gland cell line (SZ95). *J Invest Dermatol* **113**, 1011–1020, 10.1046/j.1523-1747.1999.00771.x (1999).

As such, in the Methods section under the subheading ‘Cell culture’,

“The human HaCaT keratinocytes were purchased from Thermo Scientific (MA, USA) and SZ95 sebocytes were procured as a gift from Dr. Christos C. Zouboulis (Dessau Medical Center; Germany).”

now reads:

“The human HaCaT keratinocytes were purchased from Thermo Scientific (MA, USA) and SZ95 sebocytes were characterized as previously described^47^.”

These errors have been corrected in the PDF and HTML versions of the Article, and in the accompanying Supplementary Materials file.

