# Supplementary material for: Inhibitory effects of superoxide dismutase 3 on Propionibacterium acnes-induced skin inflammation
Source: Sci Rep. 2018 Mar 5;8:4024. doi: 10.1038/s41598-018-22132-z (PMC5838256; doi:10.1038/s41598-018-22132-z)
Supplement: Supplementary file 1 — Supplementary Materials [file 41598_2018_22132_MOESM1_ESM.pdf]

Supplementary Materials for

Inhibitory effects of superoxide dismutase 3 on *Propionibacterium acnes*-induced skin inflammation

Cuong Thach Nguyen[1], Shyam Kishor Sah[1], Christos C. Zouboulis[2], Tae-Yoon Kim[1]

[1]Department of Dermatology, College of Medicine, The Catholic University of Korea, Seoul 137-040, South Korea.

[2]Departments of Dermatology, Venereology, Allergology and Immunology, Dessau Medical Center, Brandenburg Medical School Theodor Fontane, 06847 Dessau, Germany

This PDF file includes:

Figs. S1 to S6

Figure legends for Figs. S1 to S6

Supp Fig S1

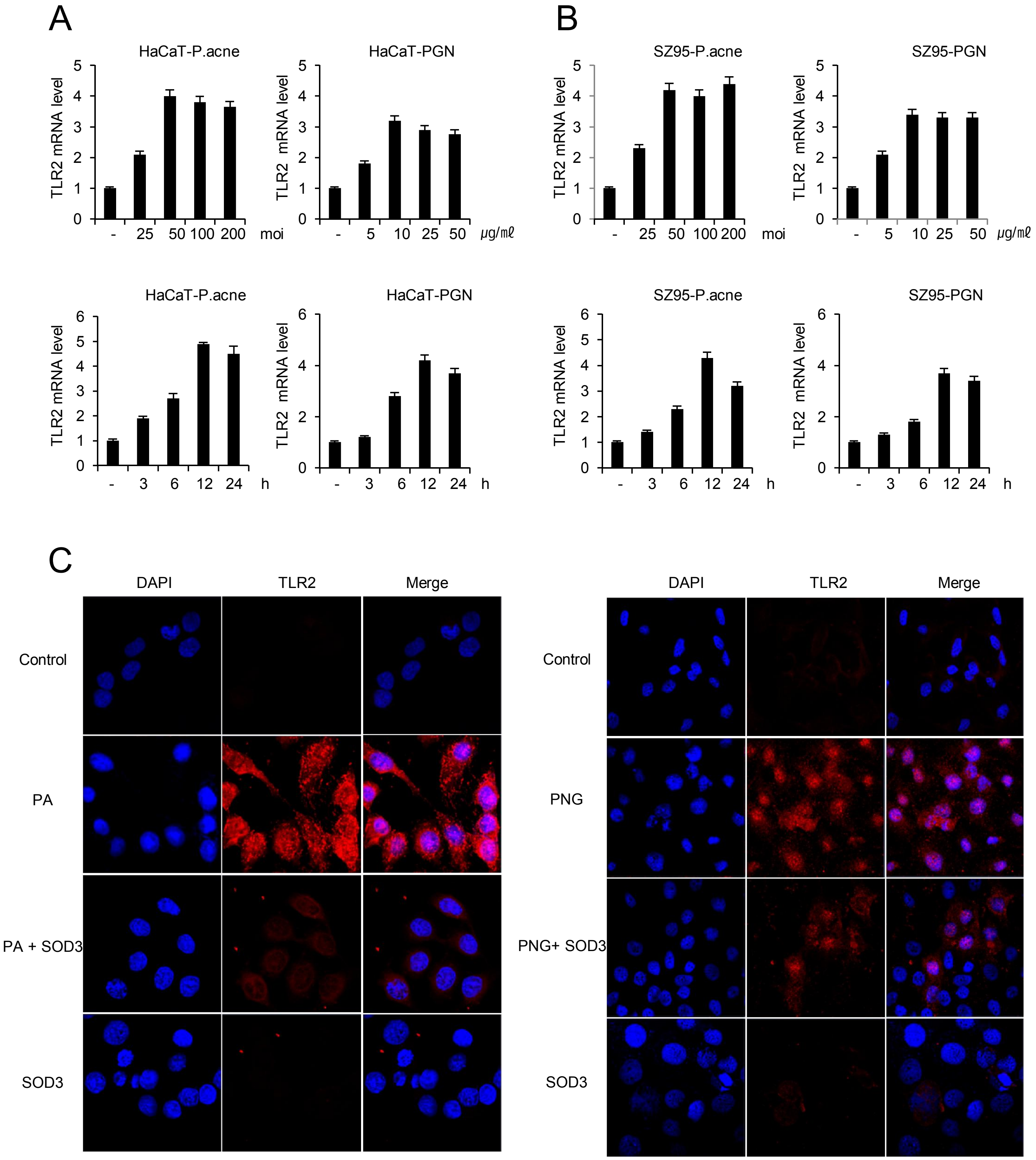
A
B
HaCaT-P.acne
HaCaT-PGN
SZ95-P.acne
SZ95-PGN
TLR2 mRNA level
- 25 50 100 200 moi
- 5 10 25 50 μg/mℓ
- 3 6 12 24 h
C
DAPI
TLR2
Merge
Control
PA
PA + SOD3
SOD3
PNG
PNG+ SOD3

Supp Fig. S2

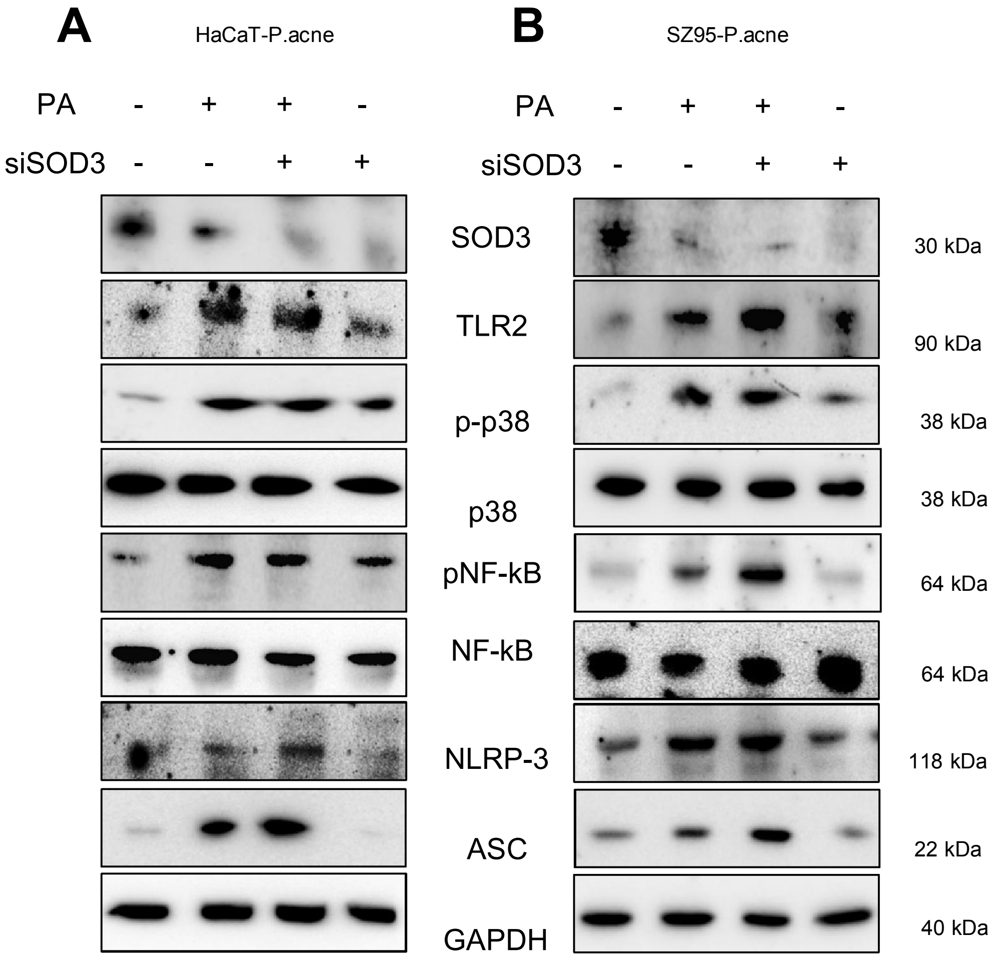
A
HaCaT-P.acne
B
SZ95-P.acne
PA - + + -
siSOD3 - - + +
PA - + + -
siSOD3 - - + +
SOD3
TLR2
p-p38
p38
pNF-kB
NF-kB
NLRP-3
ASC
GAPDH
30 kDa
90 kDa
38 kDa
38 kDa
64 kDa
64 kDa
118 kDa
22 kDa
40 kDa

Supp Fig S3

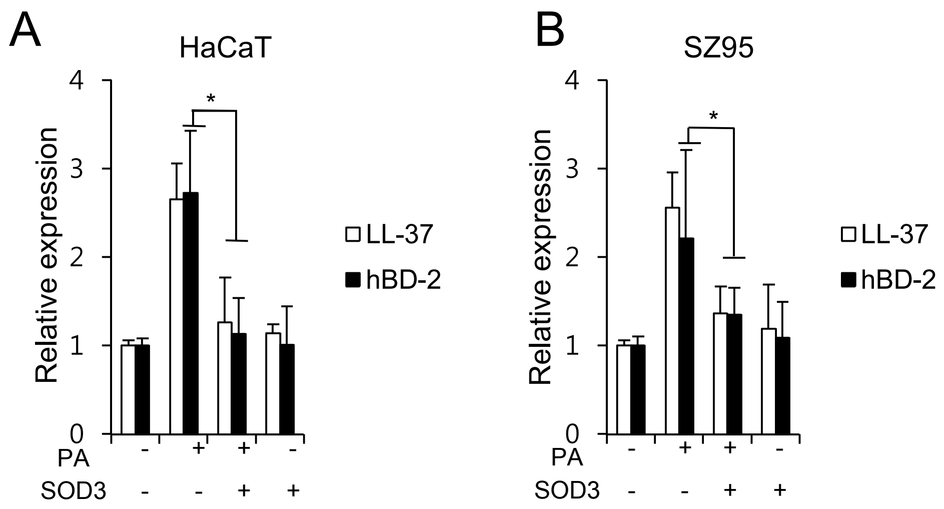

Supp Fig S4

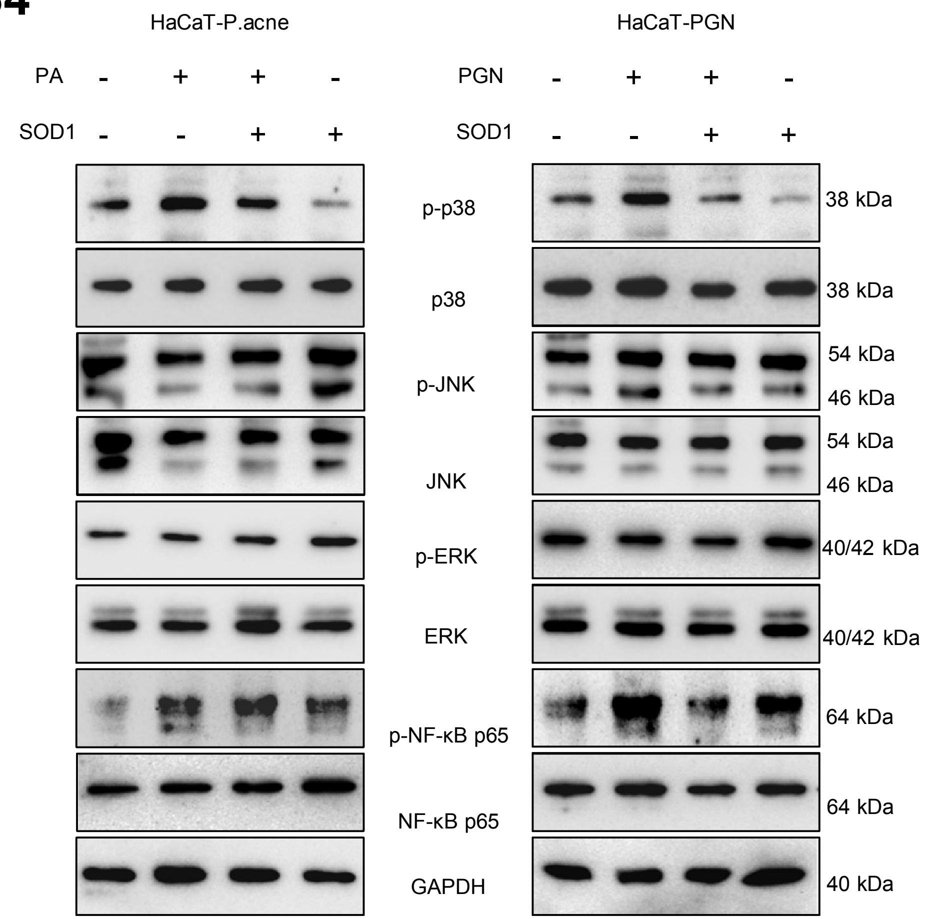
HaCaT-P.acne
HaCaT-PGN
PA - + + -
SOD1 - - + +
PGN - + + -
SOD1 - - + +
p-p38
p38
p-JNK
JNK
p-ERK
ERK
p-NF-κB p65
NF-κB p65
GAPDH
38 kDa
38 kDa
54 kDa
46 kDa
54 kDa
46 kDa
40/42 kDa
40/42 kDa
64 kDa
64 kDa
40 kDa

Supp Fig S5

A. Band density of Fig. 1

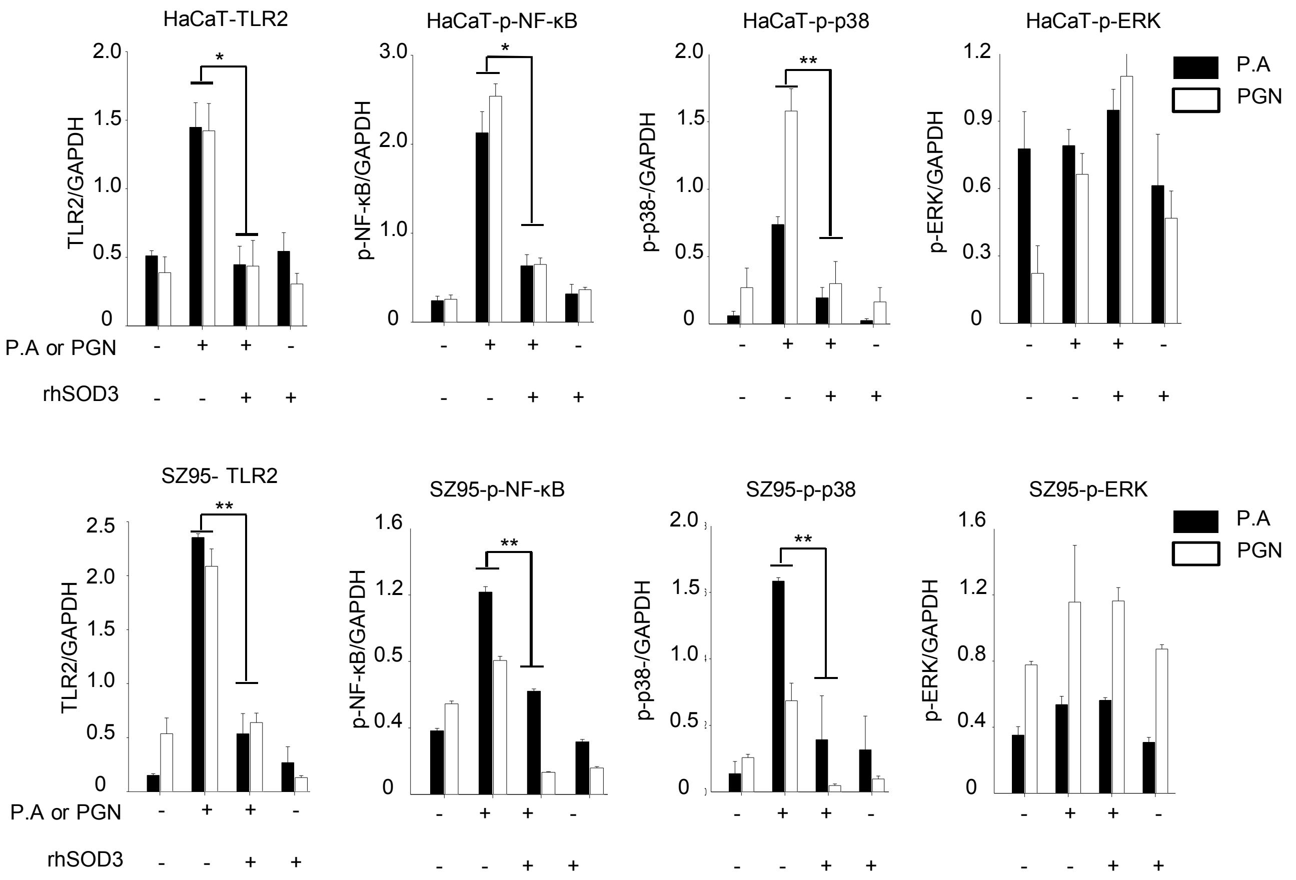

B. Band density of Fig. 2

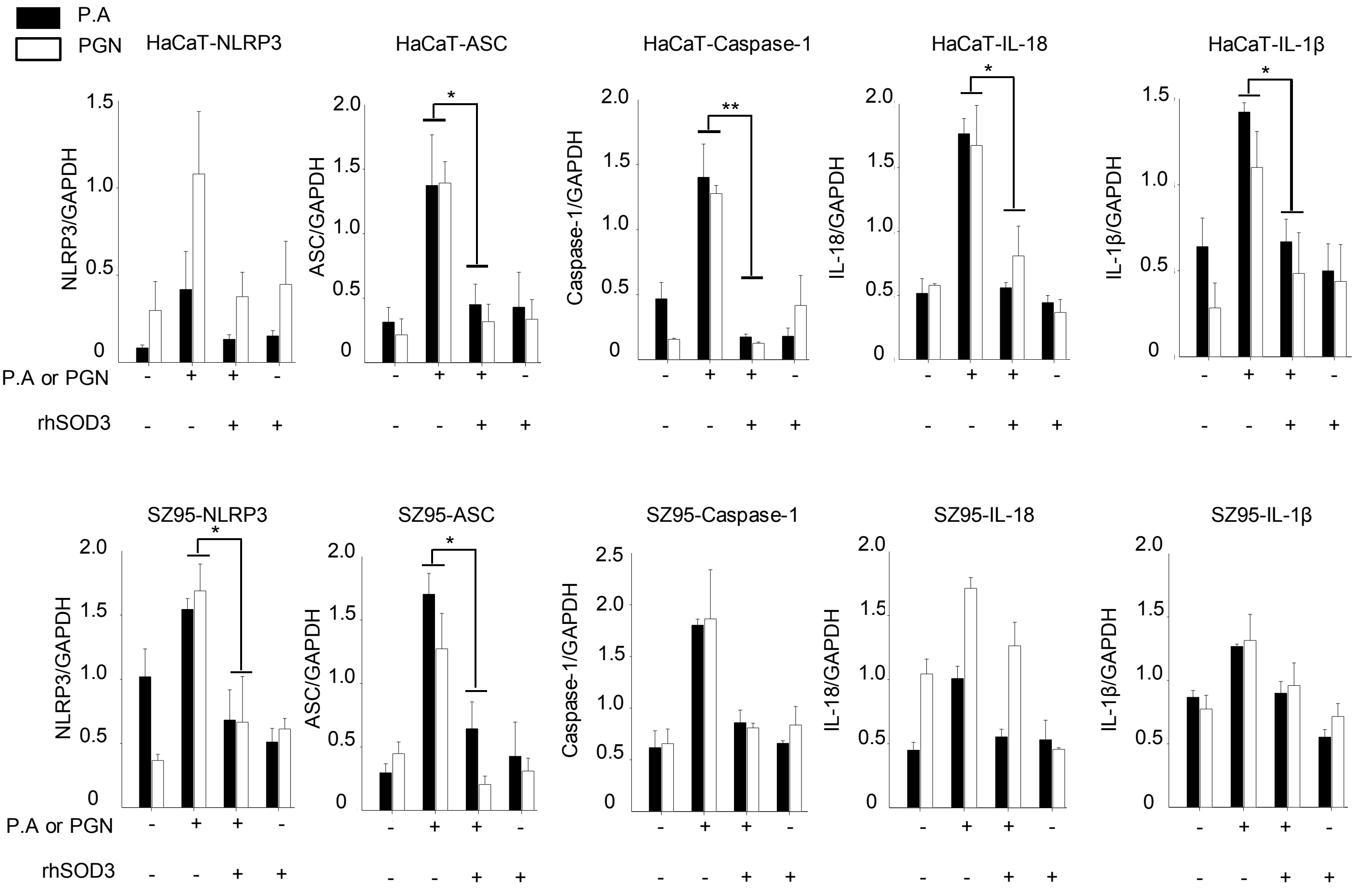

C. Band density of Fig. 6

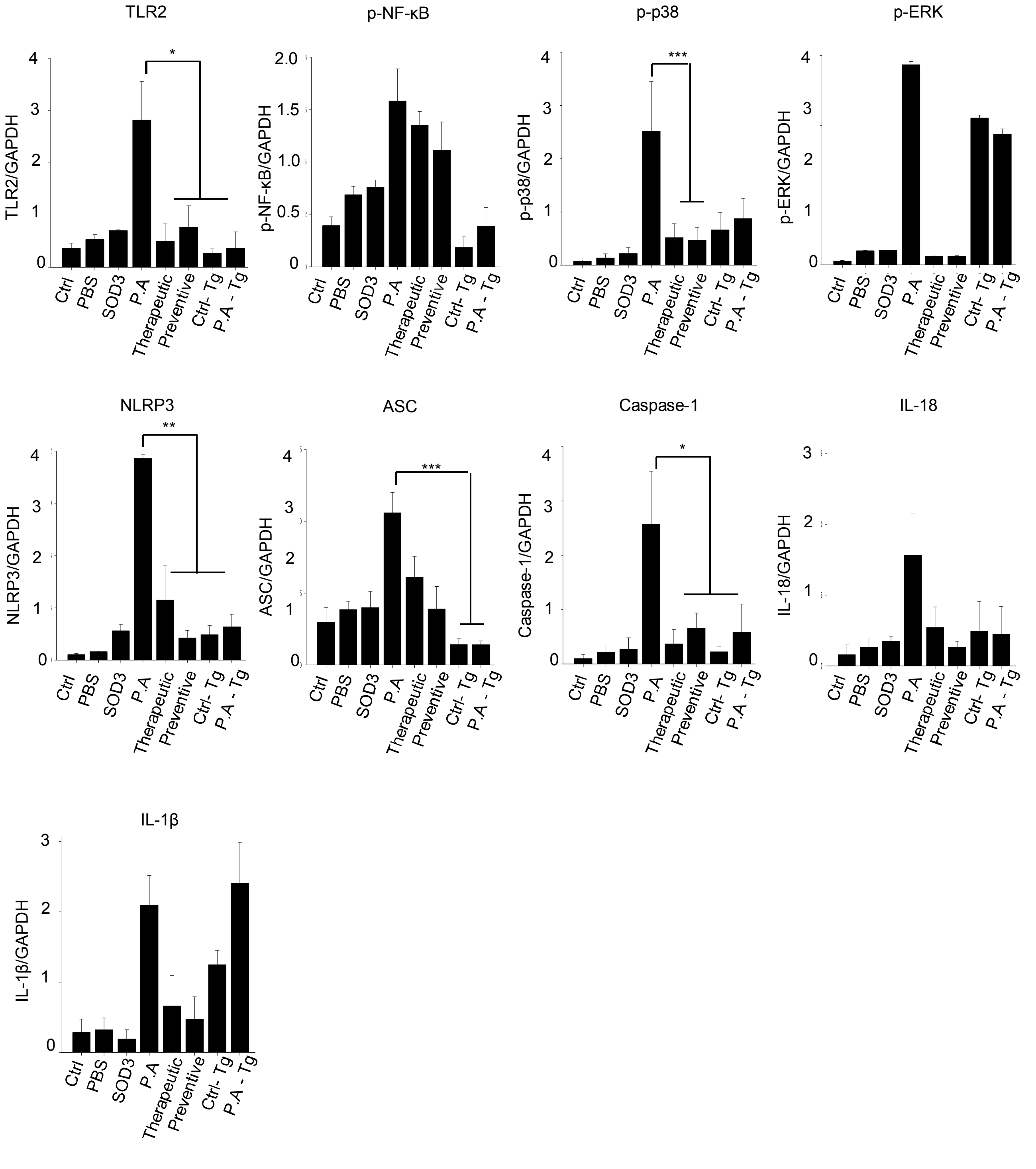

D. Band density of Sup. Fig. 3

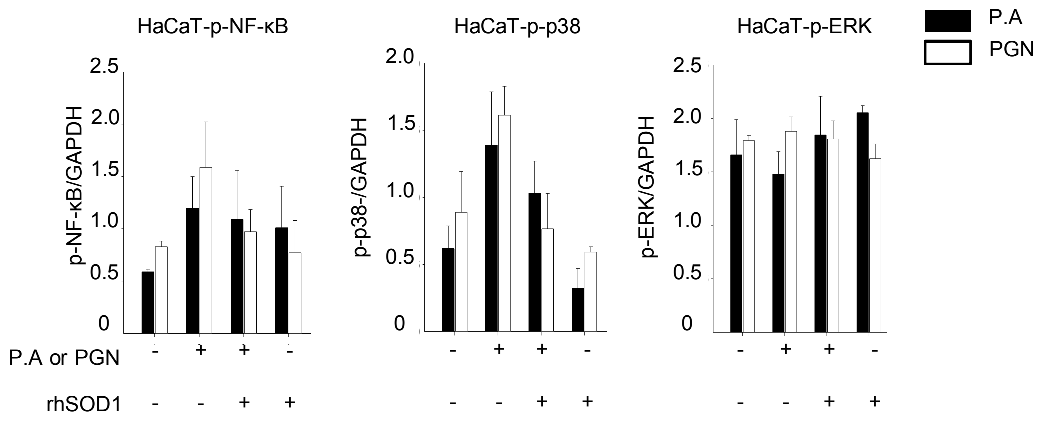

Supp Fig S6

Figure 1B

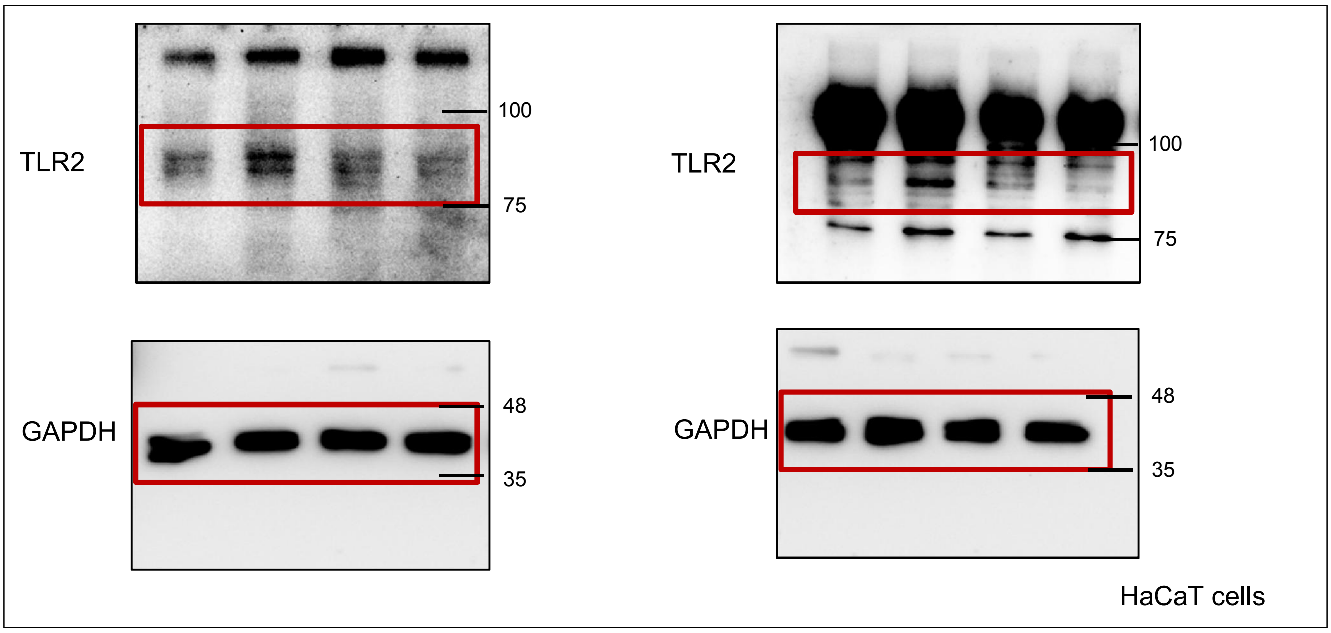

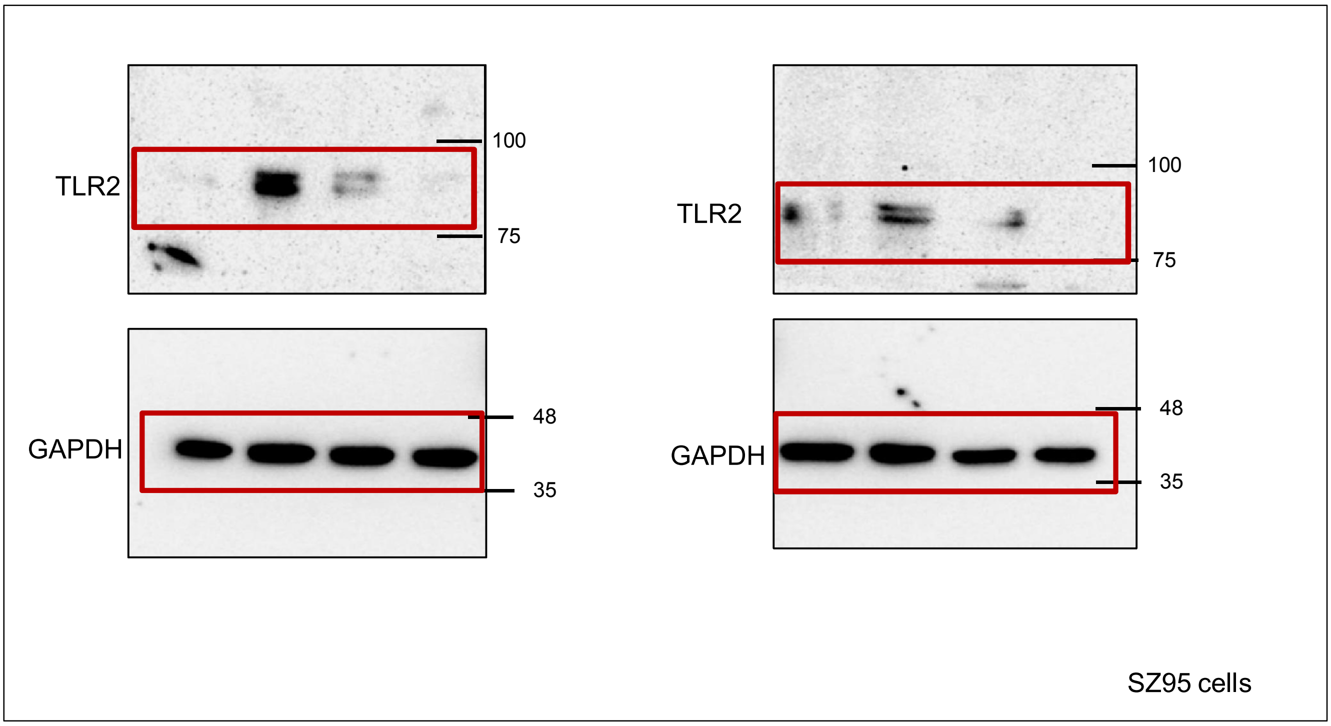

Figure 1C

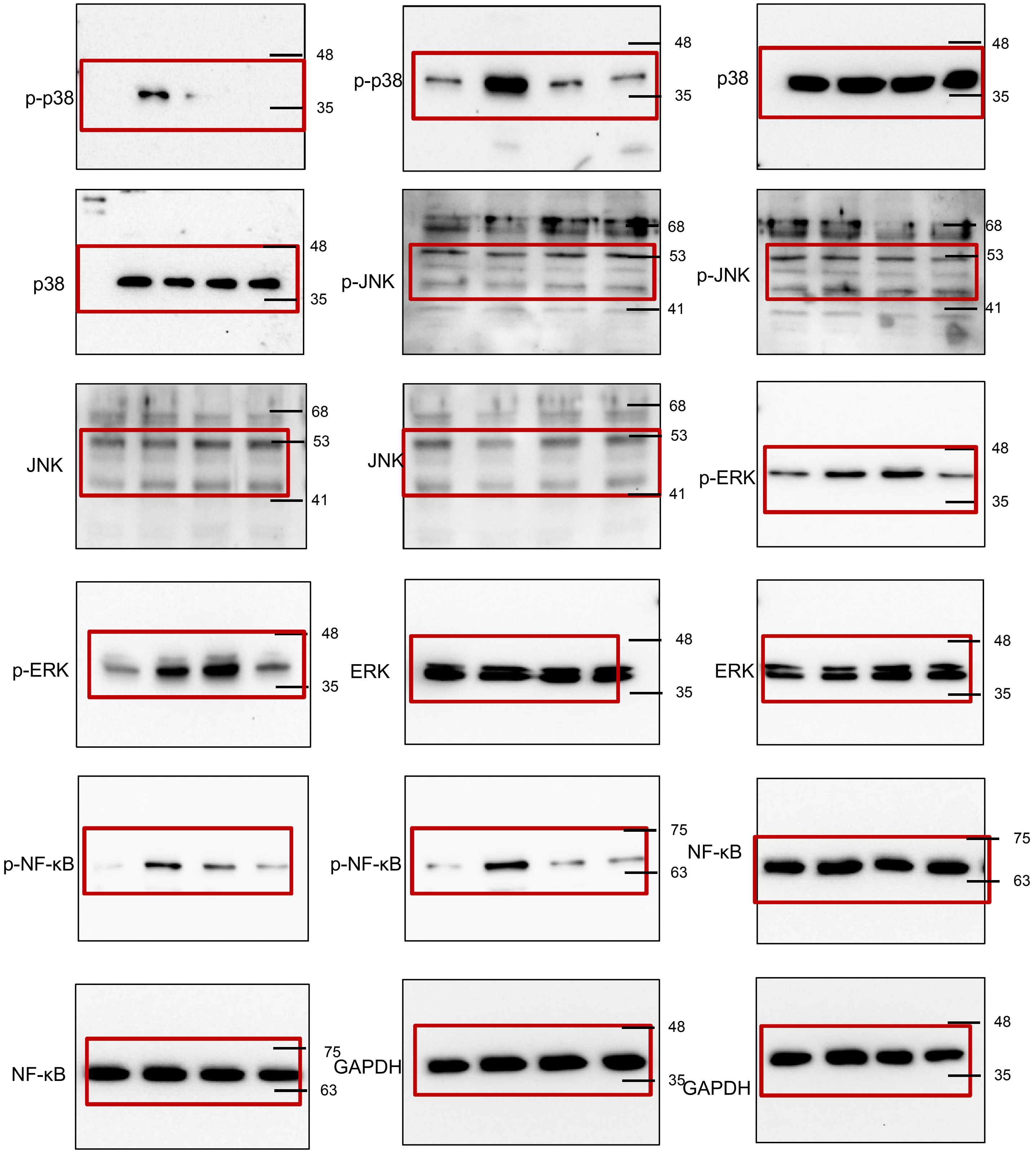
p-p38
48
35
p-p38
48
35
p38
48
35
p38
48
35
p-JNK
68
53
41
p-JNK
68
53
41
JNK
68
53
41
JNK
68
53
41
p-ERK
48
35
p-ERK
48
35
ERK
48
35
ERK
48
35
p-NF-κB
p-NF-κB
75
63
NF-κB
75
63
NF-κB
75
63
GAPDH
48
35
GAPDH
48
35

Figure 1D

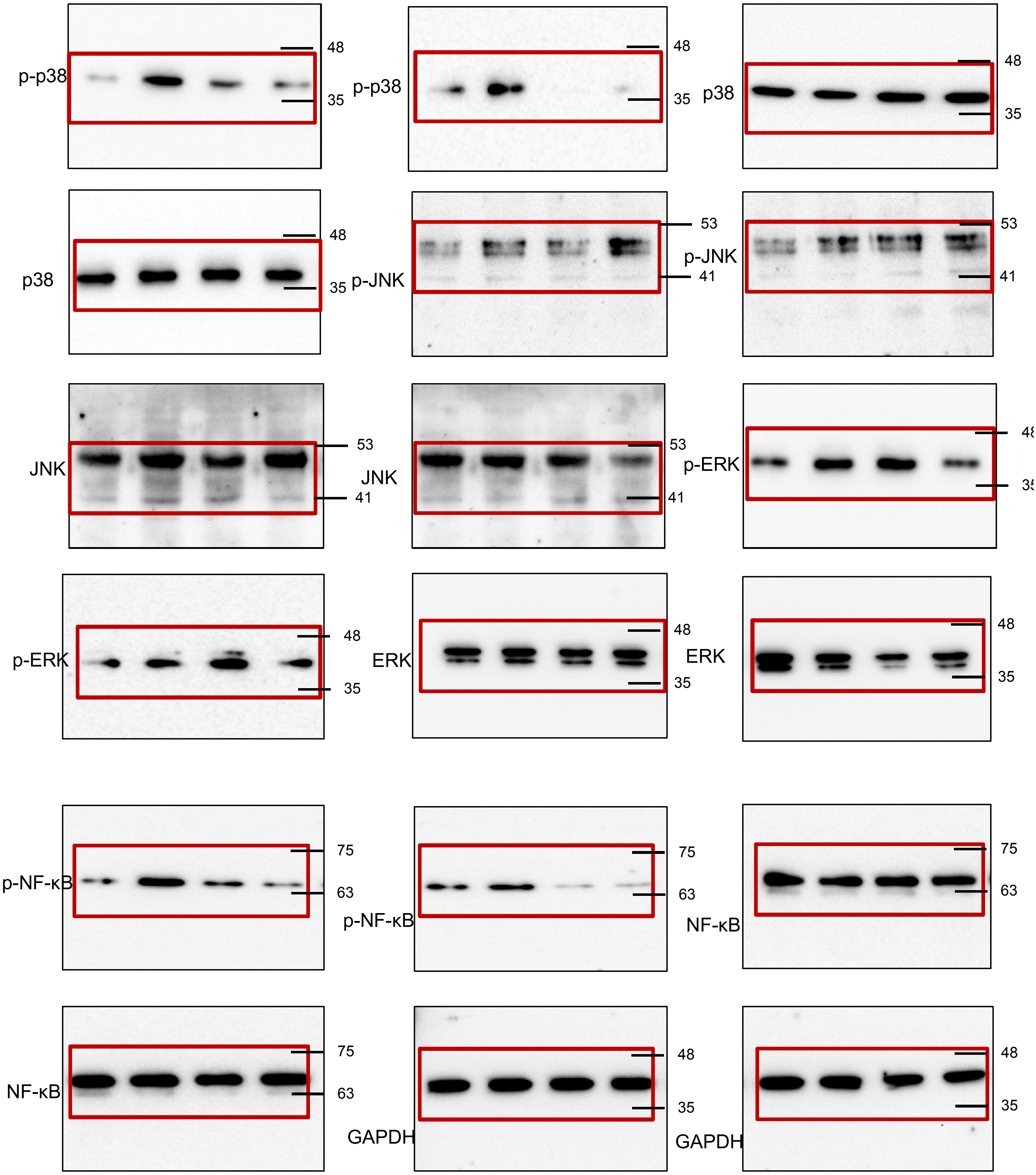
p-p38
48
35
p-p38
48
35
p38
48
35
p38
48
35
p-JNK
53
41
p-JNK
53
41
JNK
53
41
JNK
53
41
p-ERK
p-ERK
48
35
ERK
48
35
ERK
48
35
p-NF-κB
75
63
p-NF-κB
75
63
NF-κB
75
63
NF-κB
75
63
GAPDH
48
35
GAPDH
48
35

Figure 2A

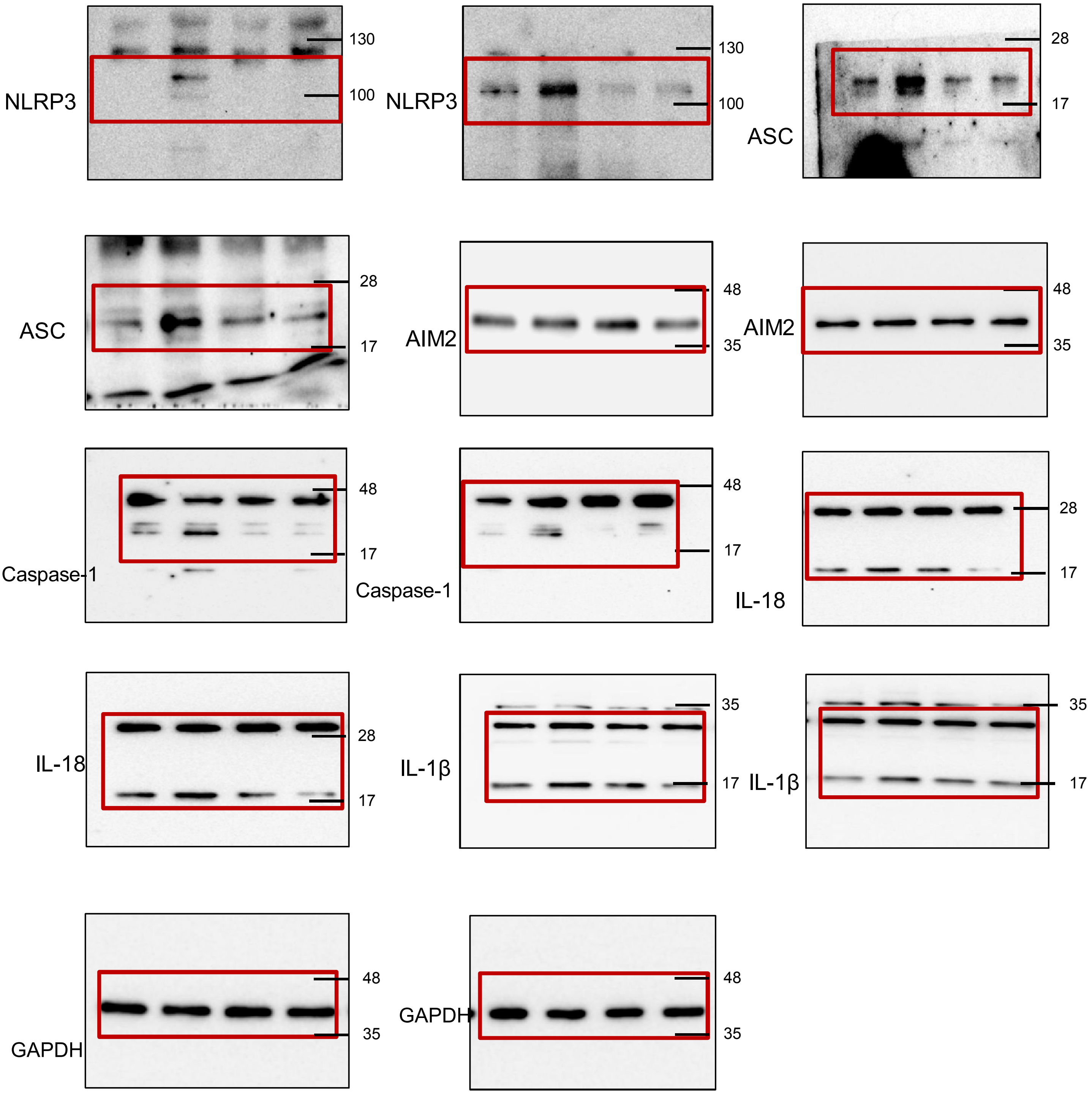
NLRP3
130
100
NLRP3
130
100
ASC
28
17
ASC
28
17
AIM2
48
35
AIM2
48
35
Caspase-1
48
17
Caspase-1
48
17
IL-18
28
17
IL-18
28
17
IL-1β
35
17
IL-1β
35
17
GAPDH
48
35
GAPDH
48
35

Figure 2B

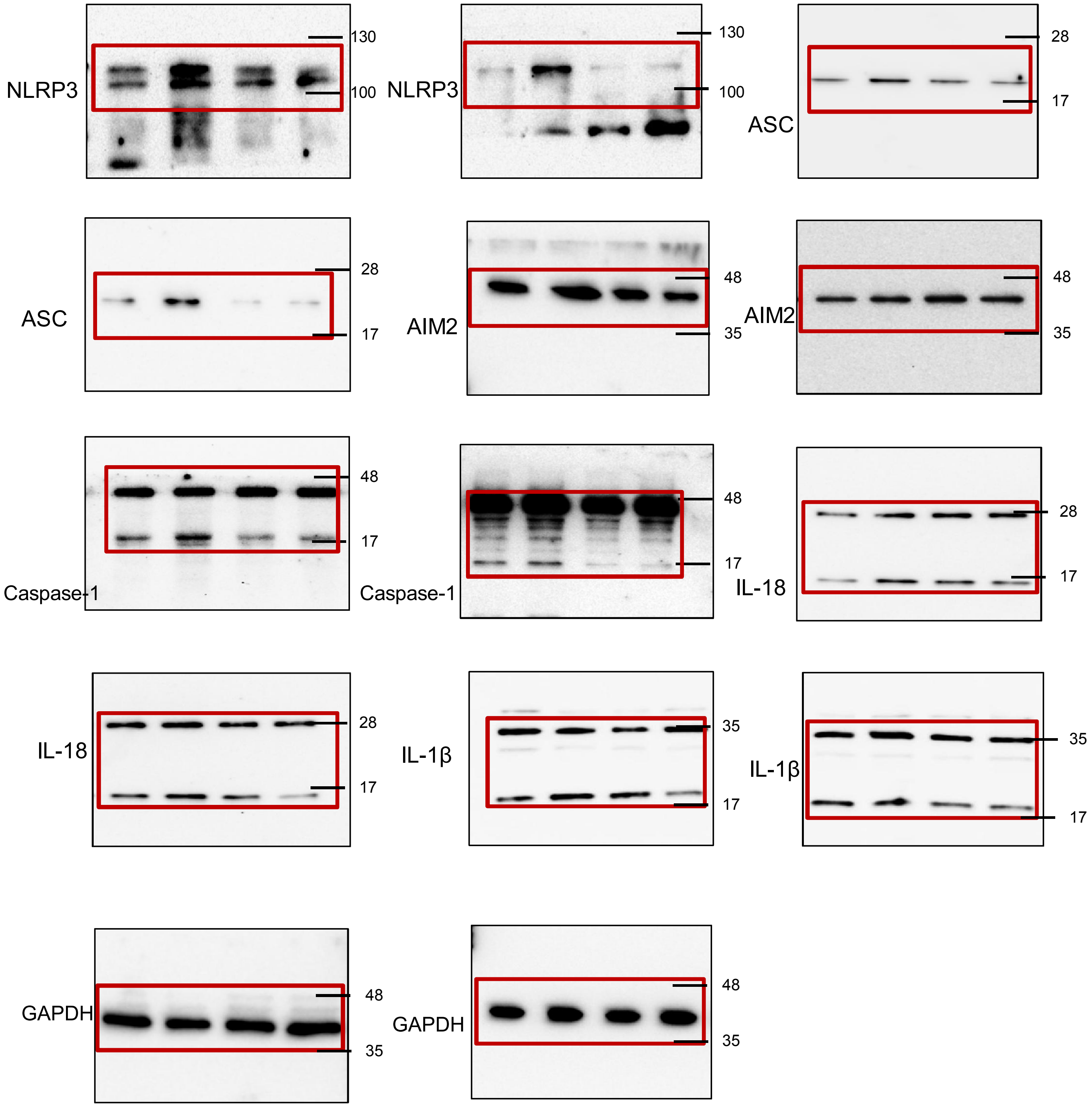
NLRP3
130
100
NLRP3
130
100
ASC
28
17
ASC
28
17
AIM2
48
35
AIM2
48
35
Caspase-1
48
17
Caspase-1
48
17
IL-18
28
17
IL-18
28
17
IL-1β
35
17
IL-1β
35
17
GAPDH
48
35
GAPDH
48
35

Figure 6A

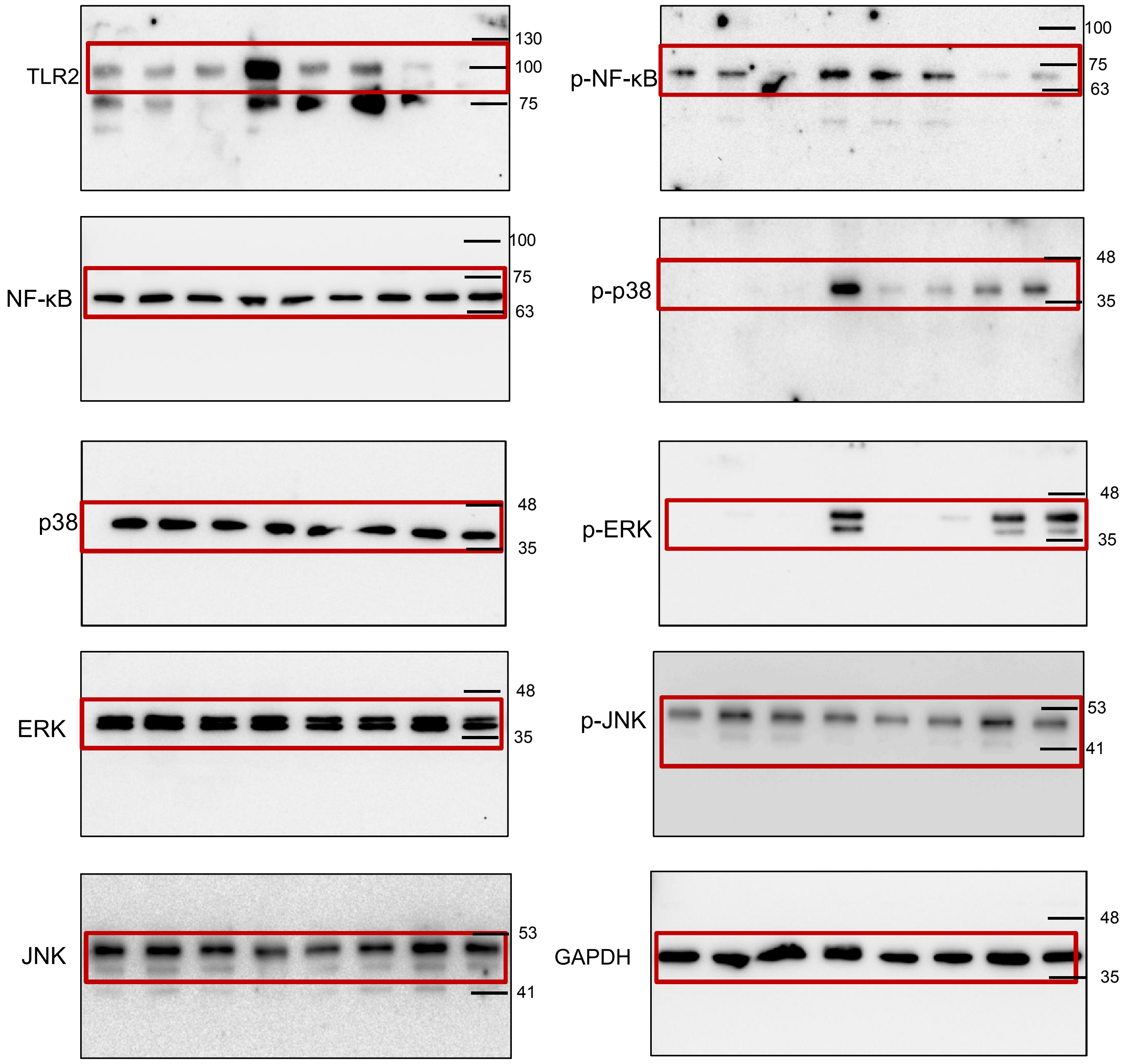

TLR2
130
100
75
p-NF-κB
100
75
63
NF-κB
100
75
63
p-p38
48
35
p38
48
35
p-ERK
48
35
ERK
48
35
p-JNK
53
41
JNK
53
41
GAPDH
48
35

Figure 6B

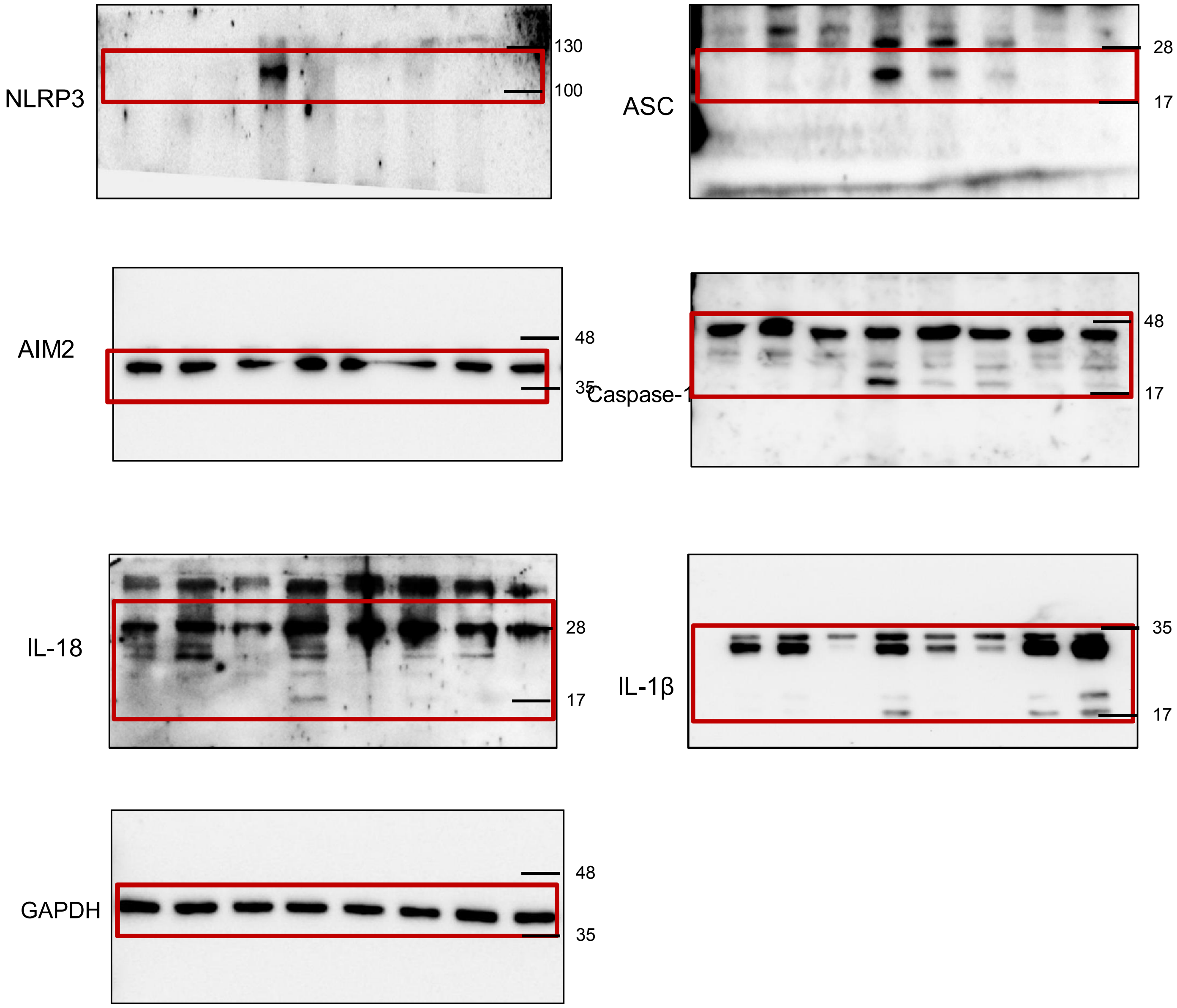
NLRP3
130
100
ASC
28
17
AIM2
48
35
Caspase-1
48
17
IL-18
28
17
IL-1β
35
17
GAPDH
48
35

Figure S2

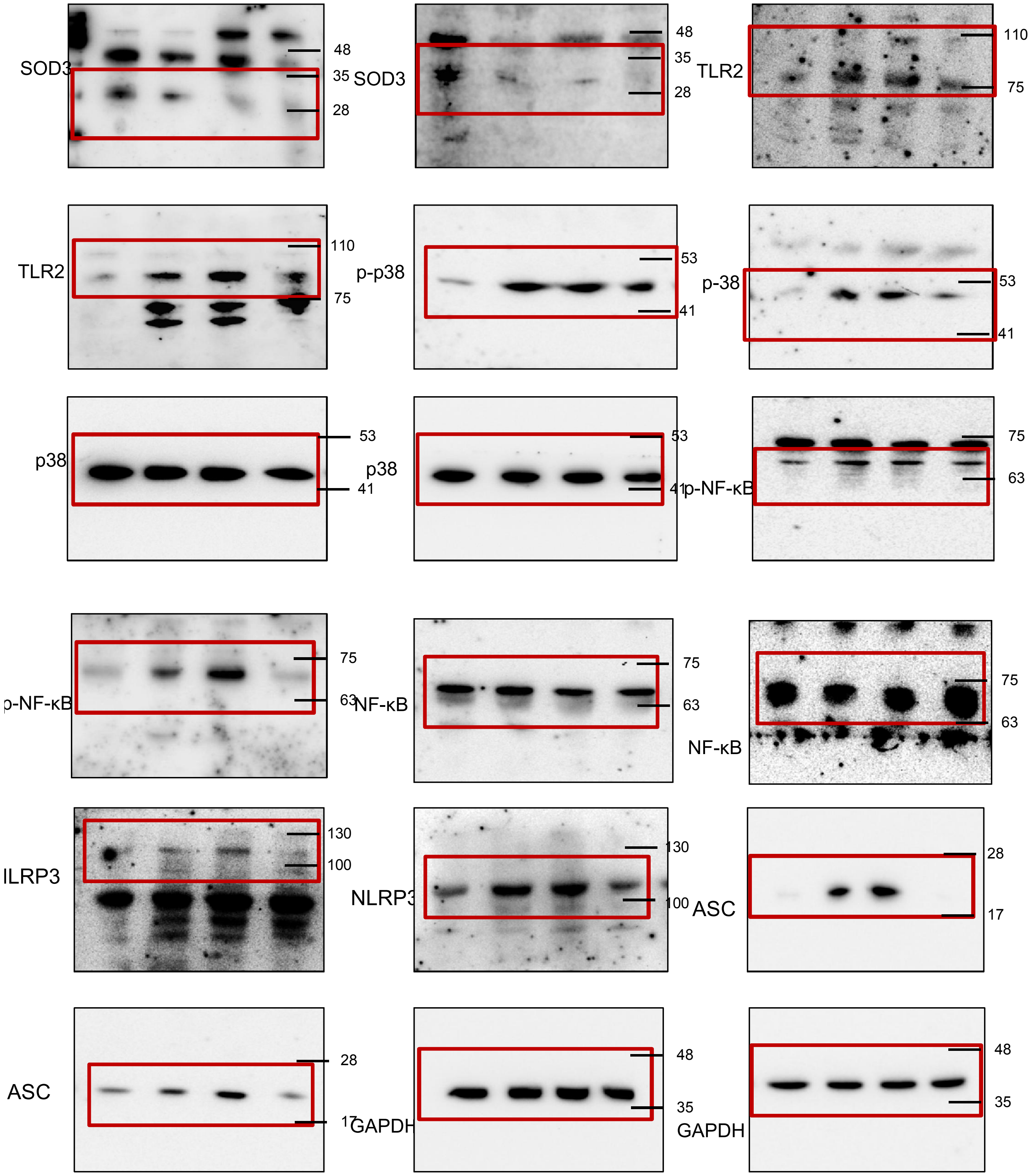

SOD3
48
35
28
SOD3
48
35
28
TLR2
110
75
TLR2
110
75
p-p38
53
41
p-38
53
41
p38
53
41
p38
53
41
p-NF-κB
75
63
p-NF-κB
75
63
NF-κB
75
63
NF-κB
75
63
NLRP3
130
100
NLRP3
130
100
ASC
28
17
ASC
28
17
GAPDH
48
35
GAPDH
48
35

Figure S4

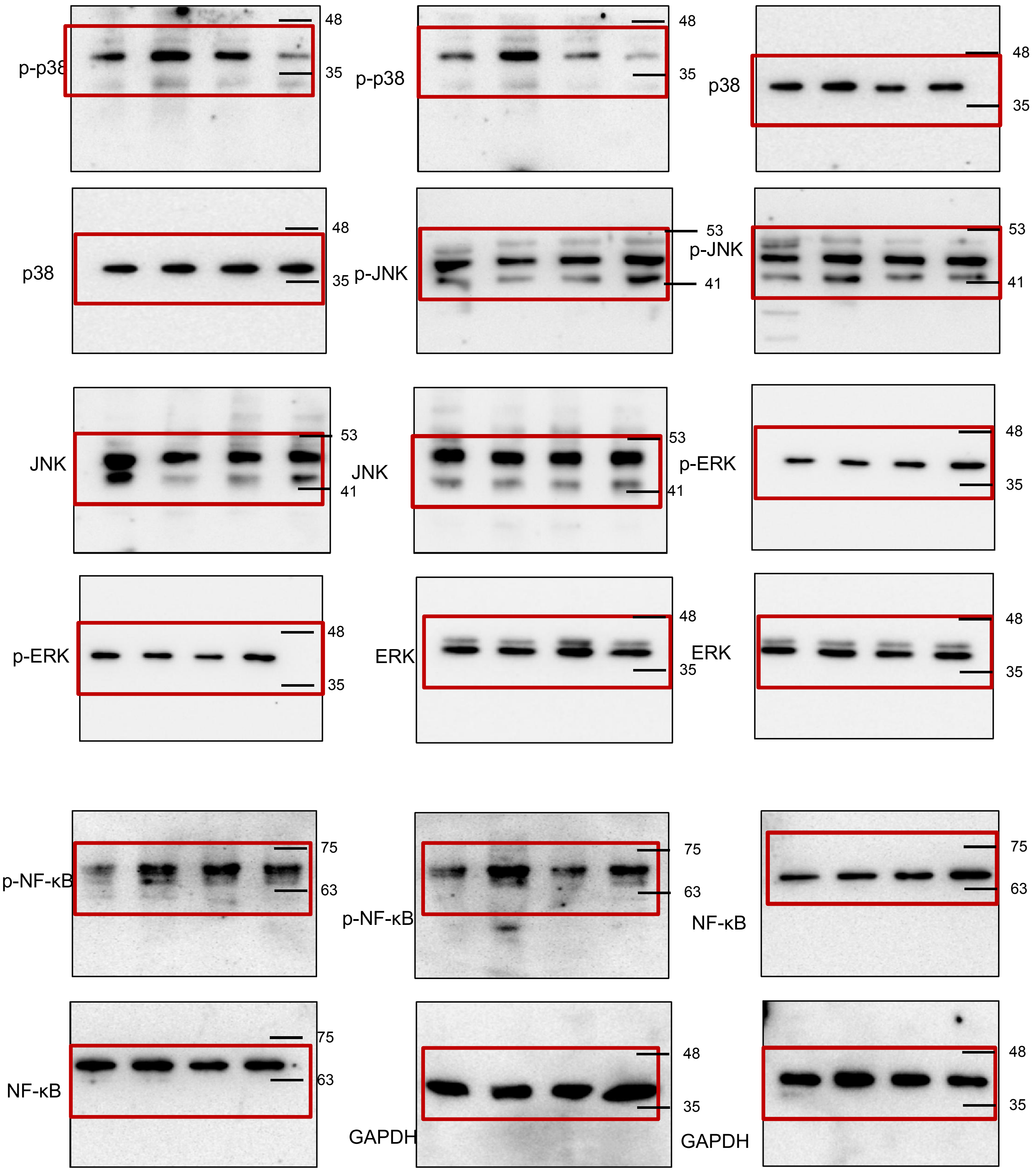

p-p38
48
35
p-p38
48
35
p38
48
35
p38
48
35
p-JNK
53
41
p-JNK
53
41
JNK
53
41
JNK
53
41
p-ERK
48
35
p-ERK
48
35
ERK
48
35
ERK
48
35
p-NF-κB
75
63
p-NF-κB
75
63
NF-κB
75
63
NF-κB
75
63
GAPDH
48
35
GAPDH
48
35

SUPPLEMENT FIGURE AND LEGENDS

Supplementary Figure 1: Expression of TLR-2 in *P. acnes* treated HaCaT and SZ95 cells. (A) HaCaT and (B) SZ95 cells were infected with heat-killed *P. acnes* (25, 50, 100, 200 MOI) or peptidoglycan (5, 10, 25, 50 μg/mL) in a time-dependent manner. Expression of TLR-2 was analyzed by qRT-PCR. (C) HaCaT cells were pretreated with SOD3 (200 U/mL) and incubated with heat-killed *P. acnes* (100 MOI) or peptidoglycan (10 μg/mL) for 24 h. Expression of TLR-2 was analyzed by confocal microscope. All data represent mean ± S.D. of three independent experiments *P<0.05, **P<0.01, ***P<0.001.

Supplementary Figure 2: **Effects of SOD3 knock-down on inflammatory markers**. (A) HaCaT and SZ95 (B) cells were knocked-down with siRNA SOD3 (30 nM) for 24h and incubated with heat-killed *P. acnes* (100 MOI) or peptidoglycan (10 μg/mL) for additional 24 h. Expression of TLR-2, p-NF-κB, and MAPKs proteins were analyzed by Western blot. Full-length blots are presented in Supplementary Figure S6. All data represent of three independent experiments.

Supplementary Figure 3: SOD3 inhibited the expression of LL-37 and hBD-2 in *P. acnes* treated HaCaT and SZ95 cells. (A) HaCaT and SZ95 (B) cells were pretreated with SOD3 (200 U/mL) and incubated with heat-killed *P. acnes* (100 MOI) or peptidoglycan (10 μg/mL) for 24 h. Expression of LL-37 and hBD-2 were analyzed by qRT-PCR. All data represent the mean ± S.D. of three independent experiments *P<0.05, **P<0.01, ***P<0.001.

Supplementary Figure 4: SOD1 did not inhibit p-NF-κB and p-p38 expression in HaCaT cells. HaCaT cells were pretreated with SOD1 (200 U/mL) and incubated with heat-killed *P.*

acnes (100 MOI) or peptidoglycan (10 μg/mL) for 24 h. Expression of p-NF-κB and MAPK proteins were analyzed by Western blot. Band density of Western blot data is shown in Supplemental Figure S5D. Full-length blots are presented in Supplementary Figure S6. All data represent of three independent experiments.

Supplementary Figure 5: **Protein band density of western blot data**. ImageJ was used to measure the band density of Western blot data from Fig. 1, Fig. 2, Fig. 6, and Fig. S3. All data represent the mean ± S.D. of three independent experiments *P<0.05, **P<0.01, ***P<0.001

Supplementary Figure 6: Full-length blots of western blot data.
